# Associations Between Extreme Temperatures and Cardiovascular Cause-Specific Mortality: Results From 27 Countries

**DOI:** 10.1161/CIRCULATIONAHA.122.061832

**Published:** 2022-12-12

**Authors:** Barrak Alahmad, Haitham Khraishah, Dominic Royé, Ana Maria Vicedo-Cabrera, Yuming Guo, Stefania I. Papatheodorou, Souzana Achilleos, Fiorella Acquaotta, Ben Armstrong, Michelle L. Bell, Shih-Chun Pan, Micheline de Sousa Zanotti Stagliorio Coelho, Valentina Colistro, Tran Ngoc Dang, Do Van Dung, Francesca K. De’ Donato, Alireza Entezari, Yue-Liang Leon Guo, Masahiro Hashizume, Yasushi Honda, Ene Indermitte, Carmen Íñiguez, Jouni J.K. Jaakkola, Ho Kim, Eric Lavigne, Whanhee Lee, Shanshan Li, Joana Madureira, Fatemeh Mayvaneh, Hans Orru, Ala Overcenco, Martina S. Ragettli, Niilo R.I. Ryti, Paulo Hilario Nascimento Saldiva, Noah Scovronick, Xerxes Seposo, Francesco Sera, Susana Pereira Silva, Massimo Stafoggia, Aurelio Tobias, Eric Garshick, Aaron S. Bernstein, Antonella Zanobetti, Joel Schwartz, Antonio Gasparrini, Petros Koutrakis

**Affiliations:** 1 Environmental Health Department (B.Alahmad, A.Z., J.S., P.K.), Harvard T.H. Chan School of Public Health, Boston, MA.; 2 Department of Epidemiology (S.I.P.), Harvard T.H. Chan School of Public Health, Boston, MA.; 3 Center for Climate, Health and the Global Environment (A.S.B.), Harvard T.H. Chan School of Public Health, Boston, MA.; 4 Environmental and Occupational Health Department, Faculty of Public Health, Kuwait University, Kuwait City (B.Alahmad).; 5 Cardiology Division, University of Maryland Medical Center, University of Maryland, Baltimore (H.Khraishah).; 6 Department of Geography, University of Santiago de Compostela, Spain (D.R.).; 7 CIBER de Epidemiología y Salud Pública, Madrid, Spain (D.R., C.Í.).; 8 Institute of Social and Preventive Medicine (A.M.V-C.); 9 Oeschger Center for Climate Change Research, University of Bern, Switzerland (A.M.V-C.).; 10 Department of Public Health Environments and Society (A.M.V-C., B.Armstrong), London School of Hygiene and Tropical Medicine, UK.; 11 Centre for Statistical Methodology (A.G.), London School of Hygiene and Tropical Medicine, UK.; 12 Centre on Climate Change and Planetary Health (A.G.), London School of Hygiene and Tropical Medicine, UK.; 13 Department of Epidemiology and Preventive Medicine, School of Public Health and Preventive Medicine, Monash University, Melbourne, Australia (Y.G., S.L.).; 14 School of Health Sciences, Cyprus University of Technology, Limassol (S.A.).; 15 Department of Primary Care and Population Health, University of Nicosia Medical School, Cyprus (S.A.).; 16 Department of Earth Sciences, University of Torino, Turin, Italy (F.A.).; 17 School of the Environment, Yale University, New Haven, CT (M.L.B., W.L.).; 18 National Institute of Environmental Health Science, National Health Research Institutes, Zhunan, Taiwan (S-C.P., Y-L.L.G.).; 19 Institute of Advanced Studies (M.S.Z.S.C.), University of São Paulo, Brazil.; 20 Department of Pathology, Faculty of Medicine (P.H.N.S.), University of São Paulo, Brazil.; 21 Department of Quantitative Methods, School of Medicine, University of the Republic, Montevideo, Uruguay (V.C.).; 22 Department of Environmental Health, Faculty of Public Health, University of Medicine and Pharmacy at Ho Chi Minh City, Vietnam (T.N.D., D.V.D.).; 23 Department of Epidemiology, Lazio Regional Health Service, Rome, Italy (F.K.D’D., M.S.).; 24 Faculty of Geography and Environmental Sciences, Hakim Sabzevari University, Sabzevar, Iran (A.E., F.M.).; 25 Environmental and Occupational Medicine and Institute of Environmental and Occupational Health Sciences, National Taiwan University and National Taiwan University Hospital, Taipei (Y-L.L.G.).; 26 Department of Global Health Policy, Graduate School of Medicine, University of Tokyo, Japan (M.H.).; 27 Center for Climate Change Adaptation, National Institute for Environmental Studies, Tsukuba, Japan (Y.H.).; 28 Department of Family Medicine and Public Health, University of Tartu, Estonia (E.I., H.O.).; 29 Department of Statistics and Computational Research, Universitat de València, Spain (C.Í.).; 30 Center for Environmental and Respiratory Health Research (J.J.K.J.), University of Oulu, Finland.; 31 Medical Research Center Oulu (J.J.K.J.), University of Oulu, Finland.; 32 Biocenter Oulu (N.R.I.R., J.J.K.J.), University of Oulu, Finland.; 33 Graduate School of Public Health, Seoul National University, South Korea (H.Kim).; 34 School of Epidemiology and Public Health, Faculty of Medicine, University of Ottawa, Canada (E.L.).; 35 School of Biomedical Engineering, College of Information and Biomedical Engineering, Pusan National University, Yangsan, South Korea (W.L.).; 36 Climate, Air Quality Research Unit, School of Public Health and Preventive Medicine, Monash University, Melbourne, Australia (S.L.).; 37 Department of Environmental Health, Instituto Nacional de Saúde Dr Ricardo Jorge, Porto, Portugal (J.M.).; 38 Department of Epidemiology, Instituto Nacional de Saúde Dr Ricardo Jorge, Lisboa, Portugal (S.P.S.).; 39 Epidemiology Research Unit (EPIUnit) (J.M.), Instituto de Saúde Pública, Universidade do Porto, Portugal.; 40 Laboratory for Integrative and Translational Research in Population Health (J.M.), Instituto de Saúde Pública, Universidade do Porto, Portugal.; 41 Laboratory of Management in Science and Public Health, National Agency for Public Health of the Ministry of Health, Chisinau, Moldova (A.O.).; 42 Department of Epidemiology and Public Health, Swiss Tropical and Public Health Institute (M.S.R.), Switzerland.; 43 University of Basel (M.S.R.), Switzerland.; 44 Department of Environmental Health, Rollins School of Public Health, Emory University, Atlanta, GA (N.S.).; 45 School of Tropical Medicine and Global Health, Nagasaki University, Japan (X.S., A.T.).; 46 Department of Statistics, Computer Science and Applications G. Parenti, University of Florence, Italy (F.S.).; 47 Institute of Environmental Assessment and Water Research, Spanish Council for Scientific Research, Barcelona (A.T.).; 48 Pulmonary, Allergy, Sleep and Critical Care Medicine Section, Department of Medicine, Veterans Affairs Boston Healthcare System, Harvard Medical School, West Roxbury, MA (E.G.).; 49 Channing Division of Network Medicine, Department of Medicine, Brigham and Women’s Hospital (E.G.), Harvard Medical School, MA.; 50 Department of Pediatrics, Boston Children’s Hospital (A.S.B.), Harvard Medical School, MA.

**Keywords:** climate change, cold temperature, heart failure, heat, hot temperature, myocardial ischemia, stroke

## Abstract

Cardiovascular disease is the leading cause of death worldwide. Existing studies on the association between temperatures and cardiovascular deaths have been limited in geographic zones and have generally considered associations with total cardiovascular deaths rather than cause-specific cardiovascular deaths.

**Methods:**

We used unified data collection protocols within the Multi-Country Multi-City Collaborative Network to assemble a database of daily counts of specific cardiovascular causes of death from 567 cities in 27 countries across 5 continents in overlapping periods ranging from 1979 to 2019. City-specific daily ambient temperatures were obtained from weather stations and climate reanalysis models. To investigate cardiovascular mortality associations with extreme hot and cold temperatures, we fit case-crossover models in each city and then used a mixed-effects meta-analytic framework to pool individual city estimates. Extreme temperature percentiles were compared with the minimum mortality temperature in each location. Excess deaths were calculated for a range of extreme temperature days.

**Results:**

The analyses included deaths from any cardiovascular cause (32 154  935), ischemic heart disease (11 745 880), stroke (9 351 312), heart failure (3 673 723), and arrhythmia (670 859). At extreme temperature percentiles, heat (99th percentile) and cold (1st percentile) were associated with higher risk of dying from any cardiovascular cause, ischemic heart disease, stroke, and heart failure as compared to the minimum mortality temperature, which is the temperature associated with least mortality. Across a range of extreme temperatures, hot days (above 97.5th percentile) and cold days (below 2.5th percentile) accounted for 2.2 (95% empirical CI [eCI], 2.1–2.3) and 9.1 (95% eCI, 8.9–9.2) excess deaths for every 1000 cardiovascular deaths, respectively. Heart failure was associated with the highest excess deaths proportion from extreme hot and cold days with 2.6 (95% eCI, 2.4–2.8) and 12.8 (95% eCI, 12.2–13.1) for every 1000 heart failure deaths, respectively.

**Conclusions:**

Across a large, multinational sample, exposure to extreme hot and cold temperatures was associated with a greater risk of mortality from multiple common cardiovascular conditions. The intersections between extreme temperatures and cardiovascular health need to be thoroughly characterized in the present day—and especially under a changing climate.

**Clinical Perspective**

**What Is New?**

- This study provided evidence from what we believe is the largest multinational dataset ever assembled on cardiovascular outcomes and environmental exposures.
- Extreme hot and cold temperatures were associated with increased risk of death from any cardiovascular cause, ischemic heart disease, stroke, and heart failure.
- For every 1000 cardiovascular deaths, 2 and 9 excess deaths were attributed to extreme hot and cold days, respectively.

**What Are the Clinical Implications?**

- Extreme temperatures from a warming planet may become emerging priorities for public health and preventative cardiology.
- The findings of this study should prompt professional cardiology societies to commission scientific statements on the intersections of extreme temperature exposure and cardiovascular health.

The second half of the 20th century witnessed significant public health gains in cardiovascular disease (CVD) prevention, mainly by addressing CVD traditional risk factors, such as tobacco use, hypertension, hyperlipidemia, diet, and physical activity.^1^ Despite this, CVD remains the leading cause of death worldwide, claiming 18 million lives yearly.^2^ Expanding public health assessments towards nontraditional risk factors, such as environmental exposures, could bring new insights into CVD prevention.

Climate change has led to substantial swings in extreme ambient temperature and increases in mortality.^3^ The Global Burden of Disease Study has recently introduced nonoptimal temperatures among the leading risk factors of mortality worldwide.^4^ In addition, a recent global analysis showed that 9.4% of all deaths can be attributed to high and low nonoptimal temperatures.^5^ Both low and high ambient temperatures were shown to contribute to CVD mortality.^6–10^ However, most temperature-related mortality studies considered total death counts combining deaths from all-causes, nonaccidental causes, or all cardiovascular causes as one stratum. Previous studies also used different designs and methodological approaches and were confined to limited geographical regions and climate zones with little attention for potential confounding from other environmental factors. Analyzing associations between extreme temperatures and specific CVD outcomes can identify vulnerable subgroups to mobilize healthcare resources and inform recommendations that is specific to CVDs.

In this study, we assembled a large global database on CVD-specific causes of death across the world from the Multi-Country Multi-City (MCC) Collaborative Research Network to investigate associations between extreme temperatures and all CVD causes, ischemic heart disease, stroke, heart failure, and arrhythmia using a standardized analytic protocol.

## Methods

### Data Collection

Investigators from each country of the MCC network (http://mccstudy.lshtm.ac.uk/) were contacted to extract specific causes of CVD mortality from national and regional death registries based on *International* *Classification of* *Diseases, Ninth* (*ICD-9*) *and Tenth Revisions* (*ICD-10*) codes. We collected the statistical underlying cause of death, defined by the World Health Organization as the disease that initiated the series of events that directly led to death. The analyses to locations with available cause-specific CVD mortality data. We used daily death counts of all cardiovascular causes (*ICD-9*; 390-459, *ICD-10*; I00-I99), ischemic heart disease (*ICD-9*; 410-414, *ICD-10*; I20-I25), stroke (*ICD-9*; 430-438, *ICD-10*; I60-I69), heart failure (*ICD-9*; 428, *ICD-10*; I50), and arrhythmia (*ICD-9*; 427 [except for 427.5], *ICD-10*; I47-I49).

The MCC environmental database has been described previously.^3,11,12^ In brief, city-specific daily ambient temperatures (expressed in °C) were obtained either from meteorological stations in national and regional networks or climate reanalysis data (Table S1). The evidence suggests that, at a large global scale, reanalysis weather data is comparable to observed data obtained from monitoring station.^13^ In locations where 2 or more stations were available, the mean across all stations was computed for each day. Temperature and mortality data were available in overlapping periods ranging from as early as January 1, 1979 in Japan, to as late as December 31, 2019 in Ecuador and Paraguay. Wherever available, we also collected daily time series data of relative humidity (24-hour average, expressed as percentage) and air pollutants such as large particulate matter (ie, particulate matter with aerodynamic diameter <10 µm; 24-hour average, in µg/m^3^), fine particulate matter (particulate matter with aerodynamic diameter <2.5 µm; 24-hour average, in µg/m^3^), near surface ozone (maximum 8-hour average, in µg/m^3^), and nitrogen dioxide (24-hour average, in µg/m^3^). In general, city-specific air pollution concentrations were derived from daily measurements of 1 or more monitors in national or regional networks. The pollution data has been described in previous MCC studies.^11,12,14^ We collected values for country-level gross domestic product (GDP) per capita from the World Bank. Climate zones were assigned to each city using the Köppen-Geiger climate classification.^15^

Data was collected within the MCC Collaborative Research Network under a data sharing agreement and cannot be made publicly available. Institutional approvals were obtained by each MCC participant in their respective country. More information about institutional sources of data can be found in the supplemental material.

### Statistical Analysis

The analyses follow a 2-stage approach: (1) at each city, we estimate the association between extreme temperatures and death from a cardiovascular cause; and (2) then we pool all the risk estimates from individual cities to get an overall estimate.

In a case-crossover design, conventionally, each individual case serves as their own control to estimate the acute associations between short-term temperature exposures and CVD events; effectively eliminating potential confounding from individual characteristics such as age, gender, diet, and smoking, among others.^16^ Since we had count data, we fit conditional quasi-Poisson models for each city, including 3-way interaction terms between year, month, and day of the week as a flexible time series alternative for a case-crossover design.^17^ The temperature-mortality association in each city was modeled as a continuous bidimensional function of temperature and lag using the distributed lag nonlinear models. Consistent with previous MCC studies, the exposure–response dimension was modeled with a quadratic B-splines with 3 internal knots placed at the 10th, 75th, and 90th percentiles of temperature for each city while the lag–response dimension was modeled with a natural spline with 3 internal knots equally spaced in the log scale.^5,18^ We considered lag periods up to 14 days to allow for delayed effects and the possibility of mortality displacement, in which deaths are advanced only a few days before when they would have occurred without the exposure.

For the second stage, to pool city-specific effect estimates, we applied a novel hierarchical extended mixed-effects framework for meta-analysis for nested groups.^19^ We fit city-specific mean summer temperature, mean winter temperature, and country-level GDP per capita as fixed meta-predictors in the meta-regression. These meta-predictors account for potential effect modification by climatologic and socioeconomic factors on the temperature–mortality relationships from different locations. In the same meta-regression model, we also fitted 2-level random effects where cities are nested within country-specific climate zones, allowing cities from the same country and climate zone to borrow information from each other. Each city has its own temperature–mortality relationships allowing us to account for differences across them. We examined the heterogeneity between cities first with intercept only and then with meta-predictors and random effects using extended versions of the Cochran Q test and *I^2^* statistic.^20^ We predicted the pooled and country-level relative risks (RR) from the fixed meta-predictor model. The estimates of the temperature–mortality relationships at the city level were derived from the best linear unbiased predictions with risks modeled on continuous scale of temperature. For each cause of death, for each city and country, we empirically identified the Minimum Mortality Temperature (MMT), which is the temperature that is associated with the least mortality risk without imposing constraints on its location. The MMT is dependent on human adaptability to local climate reflecting the most comfortable, optimum temperature in a given location.^21,22^

There is evidence that people can adapt, at least partially, to the temperatures that they are used to experiencing.^23–25^ Therefore, the impact of given extreme temperature event on human health can depend on where and when it occurs. In some locations a 35°C day is a typical summer day, whereas in other locations it can cause incalculable damage. Accordingly, temperature extremes are defined by comparison to some local average (eg, the top 1% of warmest days recorded in a particular location) rather than to an absolute temperature (such as 35°C).^25^ The RR of death by CVD outcome is, therefore, reported for extreme heat as the location-specific 99th percentile temperature versus MMT, and extreme cold as the 1st percentile temperature versus the MMT. We then estimated the proportion of excess deaths that are attributable to extreme temperatures ranges. Ranges of extreme cold and hot days were also defined as location-specific days that were below the 2.5th or above the 97.5th temperature percentile, respectively.^18^ We computed empirical CIs (eCI) using Monte Carlo simulations assuming a multivariate normal distribution of the best linear unbiased predictions of reduced coefficients.

In additional analyses, we computed excess deaths associated with all hot and cold temperatures above and below the MMT. We also stratified the cities by quartiles of GDP per capita and ranked them by the relative risk for each CVD cause of death.

To assess robustness of findings, we modeled the exposure–response curves with 4 knots at the 10th, 50th, 75th, and 90th percentiles, 5 knots at the 5th, 25th, 50th, 75th, and 95th percentiles, and increased the lag period up to 21 days. We then adjusted for relative humidity, ozone, nitrogen dioxide, particulate matter with aerodynamic diameter <10 µm, and particulate matter with aerodynamic diameter <2.5 µm in cities where such data was available. We also adjusted for other temperature variables such as interday temperature variability and sustained durations of heatwaves. Last, we controlled for very slow decadal changes in mortality and/or temperature trends by including a natural spline function of time with 1 knot every 10 years. All analyses were carried out using R software (version 4.2.1) with *gnm*, *dlnm*, and *mixmeta* packages. Detailed steps of the statistical analyses are presented in the Supplemental Methods.

## Results

### Descriptive Analyses

**Figure 1.**
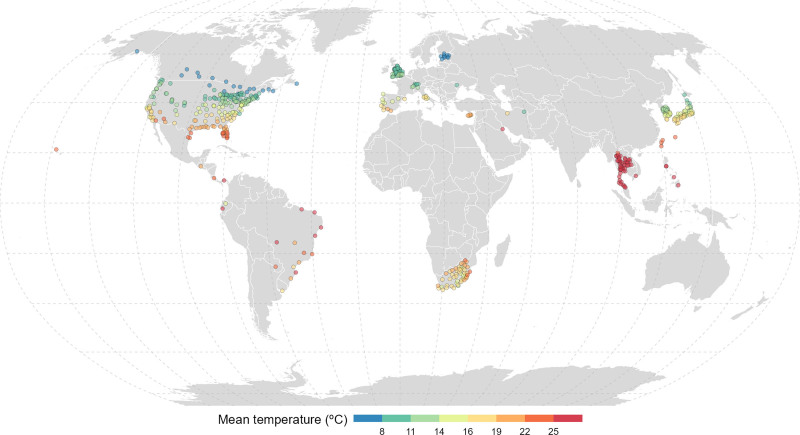
**Map of all 567 locations.** Each city is represented with average annual temperature in °C.

The analyses included 567 cities from 27 countries. We examined a total of 32 154 935 all-cause CVD deaths, 11 745 880 ischemic heart disease deaths, 9 351 312 stroke deaths, 3 673 723 heart failure deaths, and 670 859 arrhythmia deaths, covering largely overlapping years between 1979 and 2019 (average time series, 16 years per country; range, 3–36 years; Table 1). Overall temperature average varied by geographical and climate region (Figure 1). The variance of temperatures was also heterogeneous as evident by the distributions in each country (Figure S1). Countries that have temperate climates such as those in Caribbean and Central America and Southeastern Asia had a narrow range of temperatures, while the majority of other countries had a considerable variance with dual peaks of frequency for cold winters and hot summers. Across the cities, the range of temperatures varied from, for example, −30°C in Helsinki, Finland to 44°C in Kuwait City, Kuwait. Similarly, the MMT percentiles for all-cause CVD varied across countries but ranged from the 78th (Taiwan) to the 93rd (Estonia) percentiles (Table S2, Figure S1).

**Table.**
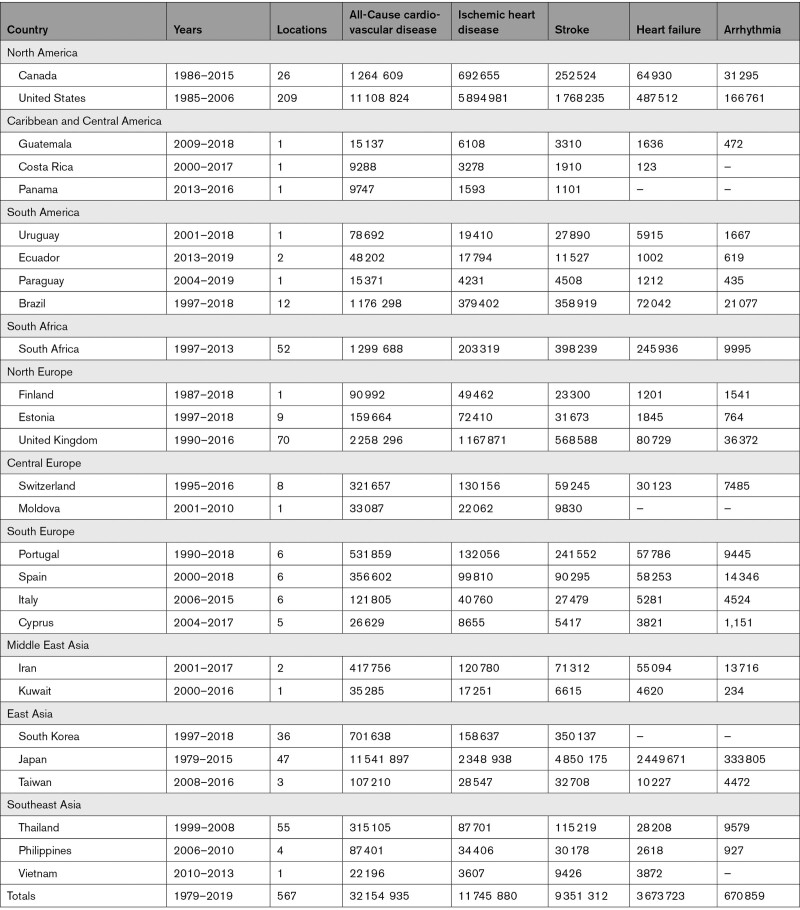
Number of Cardiovascular Cause-Specific Deaths in Each Country

The overall missing rates for data for temperature, all-cause CVD, ischemic heart disease, stroke, heart failure, and arrhythmia time series were 0.71%, 0.08%, 0.38%, 0.26%, 0.76%, and 0.24%, respectively. Detailed descriptive statistics and information on missing data are summarized in Tables S1 to S3.

### Regression Analyses

**Figure 2.**
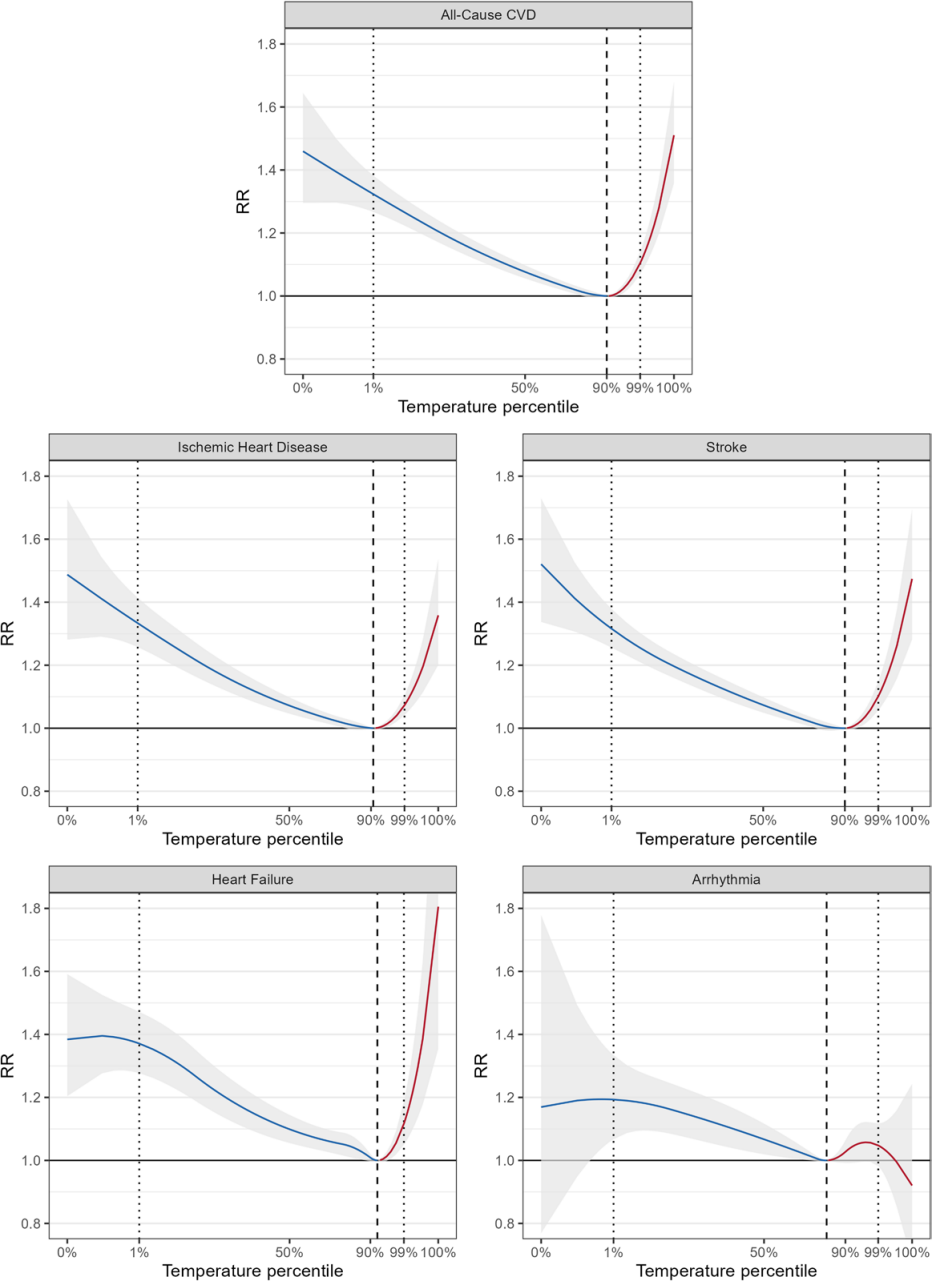
**Pooled exposure–response relationships.** Relationships are described as temperature percentiles and relative risk (RR) of different cardiovascular causes of death: all-cause cardiovascular (567 cities), ischemic heart disease (567 cities), stroke (567 cities), heart failure (524 cities), and arrhythmia (441 cities). Dashed line indicates the minimum mortality temperature. Dotted line indicates the 1st percentile (extreme cold) and the 99th percentile (extreme heat). X-axis was transformed from absolute temperatures (°C) to percentiles to enable a comparative application of the association. CVD indicates cardiovascular disease.

**Figure 3.**
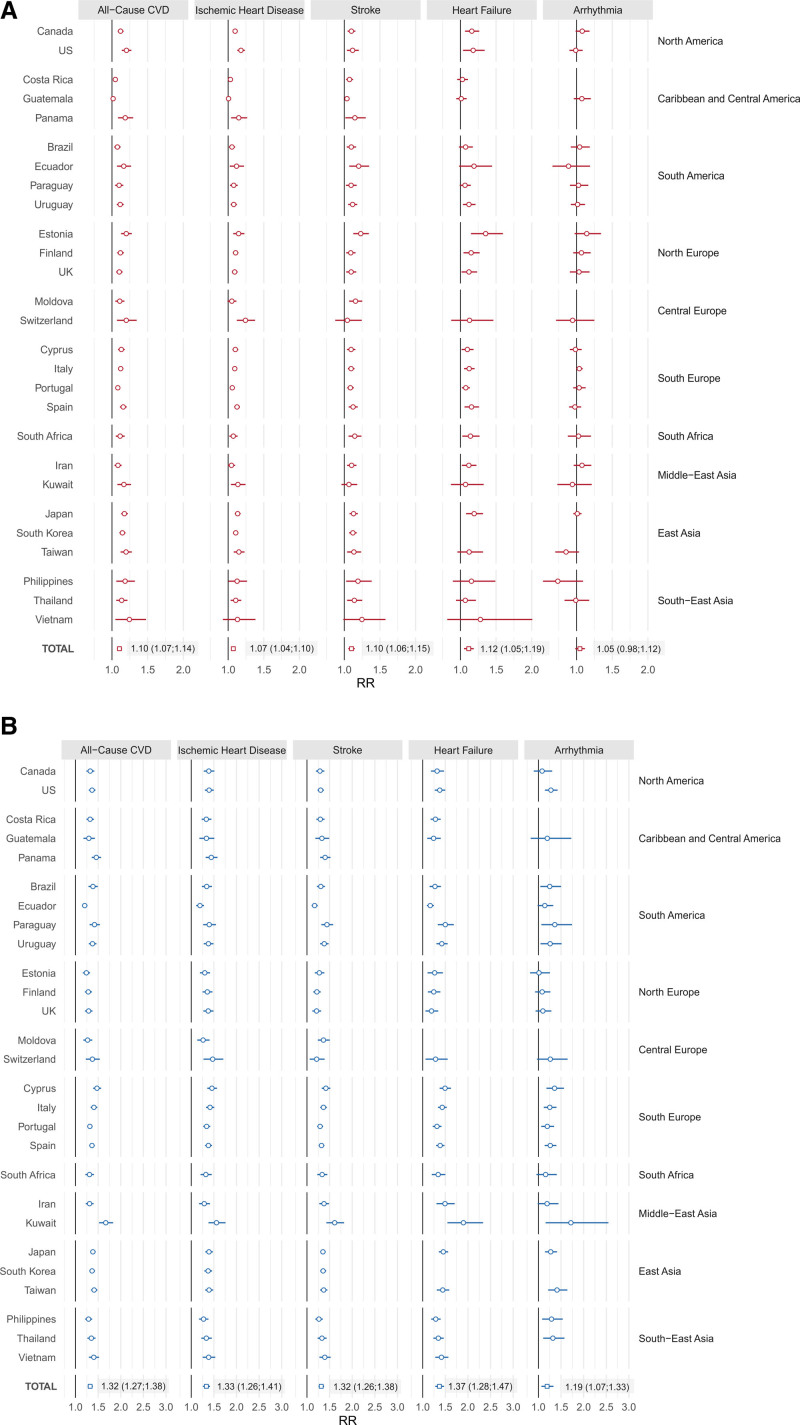
**Relative risks of death from different cardiovascular-specific causes. A**, Relative risks of death from extreme heat comparing the 99th percentile to the minimum mortality temperature in each country as well as the pooled estimate. **B**, Relative risks (RR) of death from extreme cold comparing the 1st percentile to the minimum mortality temperature in each country, as well as the pooled estimate.

The pooled overall temperature and CVD mortality relationships were non-linear with increased mortality risk in both hot and cold temperature ranges (Figure 2). The RR of death increased gradually for the cold temperatures below the MMT, while the slope for hot temperatures was slightly steeper especially with heart failure where the RRs appear to escalate quickly. The pooled RRs of death associated with extreme heat (99th percentile vs MMT) from ischemic heart disease, stroke, and heart failure were 1.07 (95% CI, 1.04–1.10), 1.10 (95% CI, 1.06–1.15), and 1.12 (95% CI, 1.05–1.19), respectively (Figure 3A). Meanwhile, the pooled RRs of death associated with extreme cold (1st percentile vs MMT) from ischemic heart disease, stroke, and heart failure were 1.33 (95% CI, 1.26–1.41), 1.32 (95% CI, 1.26–1.38), and 1.37 (95% CI, 1.28–1.47), respectively (Figure 3B). Risk of dying from arrhythmias was associated with greater uncertainty and smaller effect size estimate for extreme heat (1.05 [95% CI, 0.98–1.12]) and cold (1.19 [95% CI, 1.07–1.33]). Exposure–response curves with temperature in the absolute scale (°C) are shown in Figures S2 and S3.

**Figure 4.**
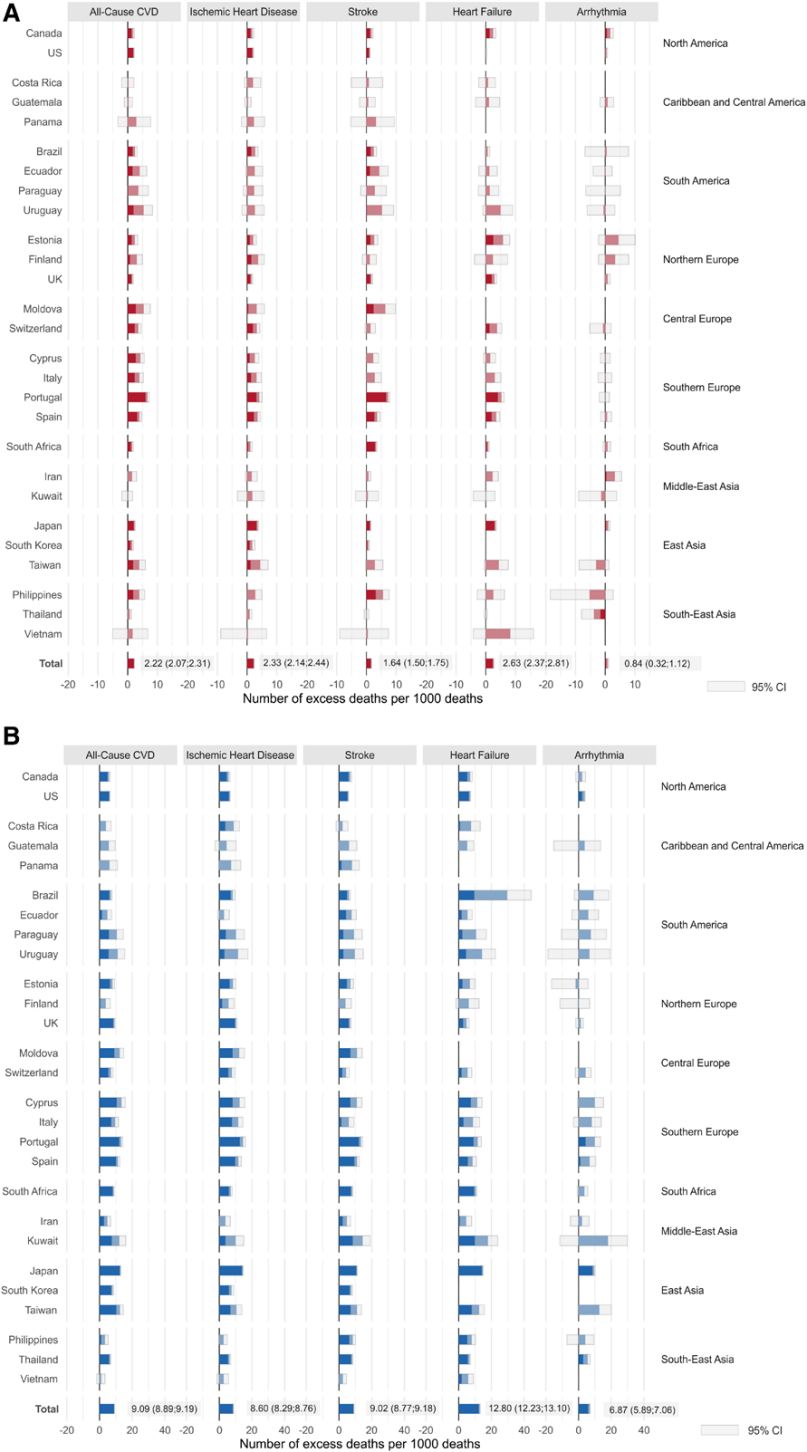
**Excess deaths from different cardiovascular-specific causes. A**, Proportion of excess deaths associated with a range of extreme hot temperatures above the 97.5th percentile, expressed as number of deaths for each 1000 cardiovascular-specific deaths in each country as well as the pooled estimate. **B**, Proportion of excess deaths associated with a range of extreme cold temperatures below the 2.5th percentile, expressed as number of deaths for each 1000 cardiovascular-specific deaths in each country as well as the pooled estimate.

For every 1000 all-cause CVD deaths, 2.2 (95% eCI, 2.1–2.3) excess deaths were attributed to extreme range of hot temperatures above the 97.5th percentile. The highest burden from extreme hot temperatures was seen for heart failure with 2.6 (95% eCI, 2.4–2.8) excess deaths for every 1000 heart failure deaths (Figure 4A). Excess mortality attributable to an extreme range of cold temperatures below the 2.5th percentile was consistently higher than heat and accounted for 9.1 (95% eCI, 8.9–9.2) for every 1000 all-cause CVD deaths and 12.8 (95% eCI, 12.2–13.1) for every 1000 heart failure deaths (Figure 4B). Country-specific risk estimates, excess deaths from extreme and all temperature ranges, and cities at highest risk are provided in the Tables S4 to S7.

Using simple models (with intercept only), the heterogeneity (*I^2^* statistic) in second-stage meta-analyses for mortality from all-cause CVD, ischemic heart disease, stroke, heart failure, and arrhythmia were 55.0%, 36.9%, 23.7%, 21.9%, and 5.2%, respectively. After including the meta-predictors (GDP per capita, mean summer and winter temperatures) and random effects (country and climate zones), the corresponding heterogeneity was reduced to 50.0%, 33.5%, 17.6%, 17.9%, and 4.5%, respectively (Table S8). Exposure–response curves stratified by GDP per capita and mean summer and winter temperatures are shown in Figures S4 to S6. For stroke and heart failure, countries with low GDP per capita seem to have higher risks of mortality compared to countries with high GDP per capita, whereas for ischemic heart disease it is the opposite (Figure S4).

The results from the main model were robust to different modelling choices and adjustments for potential time-varying confounders (Table S9). Changing the number and location of knots in the temperature exposure–response curve did not substantially change the results. Increasing the lag period from 14 to 21 days was associated with slightly higher RR and excess deaths from cold temperatures. The results were not affected by adjustments for temperature variability, heatwaves, long-term trends, relative humidity, and air pollutants (eg, ozone, nitrogen dioxide, particulate matter with aerodynamic diameter <10 µm, and particulate matter with aerodynamic diameter <2.5 µm [included 1 at a time]).

## Discussion

Our study provides evidence for an association between extreme temperatures and CVD cause-specific mortality from what we believe is the largest multinational dataset ever assembled. The data includes outcomes from countries in different climate zones, and with different socioeconomic and demographic characteristics. Previous studies reported associations for all-cause CVD mortality as an umbrella outcome but with substantial heterogeneity; this limits the ability to draw meaningful clinical conclusions about specific CVD causes of death to inform health care providers on vulnerable patients and direct public health planning. Using state-of-the-art unified analytical protocol, we examined specific causes and found considerably less heterogeneity and robust pooled-effect size estimates. Overall, extreme cold associations were found to be substantial for different causes of CVD deaths, with the largest excess deaths associated with heart failure. While hot temperatures showed steeper slopes and high RRs that increased with greater temperatures, these occurred during a small proportion of days; therefore, excess CVD deaths from sustained extreme cold were larger than those from quickly escalating heat risks.

Observed associations for temperature extremes and all-cause CVD in our study were in line with previous meta-analyses.^8–10^ Pooling across different definitions of cold and heat, 1 meta-analysis of 26 temperature–mortality studies found that cold exposure had a greater effect size on all-cause CVD mortality (pooled RR, 1.055; 95% CI, 1.050–1.060) compared to heat exposure (pooled RR, 1.013; 95% CI, 1.011–1.015).^26^ Another meta-analysis, restricted to the elderly population, found an increase in all-cause CVD mortality for every 1°C departure from the optimal temperature.^27^ The *I^2^* statistic for heterogeneity in these meta-analyses ranged from 88.2% to 98.9%. In contrast to these studies, our findings had a markedly lower *I^2^* of 50.0% after accounting for meta-predictors.

Ischemic heart disease is the commonest cause of CVD mortality, and it was the most frequent cause of death in our dataset accounting for 37% of CVD deaths. We estimated that about 1% of all ischemic heart disease deaths are attributed to extreme temperatures alone. Some have argued that future ischemic heart disease risk is expected to be more evident with increasing temperatures induced by climate change.^28^

Globally, stroke is estimated to be the third leading cause of death and disability combined in 2019.^29^ In this study, we estimate that for every 1000 stroke deaths, 1.6 and 9 excess deaths are attributed to extreme heat and cold days, respectively. While latitude, average temperatures, and sex were effect modifiers for previously examined temperature–stroke relationships,^30^ we found that GDP per capita and average summer and winter temperatures also partially explained heterogeneity. Previous studies support an association between temperature and stroke risk^31^; yet, the role of GDP per capita on temperature-related stroke deaths is not understood. The Global Burden of Disease Study showed that stroke mortality rates in low-income countries are 3× higher than high-income countries.^29^ Our effect modification analysis, albeit inconclusive, is suggestive of a potential link between GDP per capita and temperature–stroke relationship. High GDP per capita may reflect both increased ability to control temperature exposures and decreased outdoor work.

To our knowledge, little evidence exists on the risk of heart failure mortality from extreme temperatures. Some studies examined the relationship between heart failure admissions and seasonality or diurnal temperature range showing higher admissions during winter months and with greater diurnal temperature range.^32,33^ The exposure–response curves for heart failure outcomes and temperatures, however, were absent. We found heart failure mortality to have the highest burden of excess deaths for both heat and cold compared to other causes of CVD mortality. Additionally, the slope for hot temperatures beyond the MMT appears to accelerate sharply.

Few studies report on arrhythmia mortality and extreme temperatures, and findings have been inconsistent for occurrence of arrhythmia.^34–37^ This inconsistency may result from different arrhythmia end points and definitions across different studies. We did not find a significant association between extreme heat and death from arrhythmia, which may have resulted from misclassified recording of arrhythmia as a cause of death. For example, fatal ventricular arrhythmias may have occurred but often is the end result of ischemia, cardiomyopathy, or other CVD causes.

There are some postulated underlying mechanisms that could explain the CVD mortality risk associated with extreme temperatures. Both cold and heat exposures evoke a series of synchronized autonomic and cardiovascular responses that ensures core body thermal homeostasis. Those responses are often altered and exaggerated with aging and in individuals with preexisting cardiovascular risk factors, leading to CVD events.^38,39^ Heat exposure leads to increased skin blood flow which dissipates heat, as well as increased sweating which evaporates heat. This leads to potential volume depletion and sympathetic system activation, resulting in increased heart rate and inotropy to maintain stroke volume despite decreased preload.^40^ Rising core body temperature also increases metabolic state and oxygen consumption. In susceptible individuals, these cascades may lead to demand ischemia or plaque rupture.^41^ Volume depletion and insensible body water losses leads to hemoconcentration and hypercoagulable states further increasing the risk of thrombosis and myocardial ischemia.^42^ Fluid shifts can disturb electrolyte balance (specifically, potassium, magnesium), increasing risk of arrhythmias in susceptible individuals.^42^ In patients with heart failure who receive diuretics, heat may result in severe volume depletion and potentially, shock.^43^ On the other hand, cold exposure leads to increased sympathetic activity that vasoconstricts skin and increases skeletal muscle tone to generate and conserve heat. This leads to catecholamine-driven rise in blood pressure (mainly through an increase in peripheral vascular resistant), and hence, increased cardiac oxygen demand to overcome this increase in afterload.^38^ Cold also leads to increased cholesterol crystals deposition in atherosclerotic plaques, rendering susceptible individuals to plaque rupture and myocardial infarction.^39^ Last, hypothermia is known to induce hypercoagulability because of increased viscosity and hemoconcentration from fluid shifting into extravascular space and clotting factor abnormalities.^39^

Climate change produces both hotter summers and colder winters, rendering populations not accustomed to these unusual weather conditions vulnerable, especially in low-income areas where there may be less adaptability to changing conditions. From a clinical perspective, the extent to which healthcare providers who treat CVD patients understand and act to minimize the risks of extreme temperatures is not fully understood. A search of all American Heart Association and European Society of Cardiology journals finds no official statements related to guidance for providers on preventing adverse outcomes from extreme temperature exposures. From a public health standpoint, evidence suggests that targeted interventions, such as warning systems for vulnerable subpopulations, may be needed to effectively prevent CVD deaths.^41^

This study has several limitations. Readers must be cautioned to interpret our findings as global estimates since some regions (especially low-income countries) were underrepresented in our data such as South Asia, the Middle East, and Africa. Different populations have markedly different characteristics, baseline rates of CVD, and are exposed to a wide range of climates. Even within the studied countries, differences in population characteristics, urbanization, health care infrastructure and housing characteristics, warrant further investigation. More specifically, socioeconomic determinants are particularly important to understand the effects that extreme temperatures might have on socially and economically vulnerable subpopulations. While we account for GDP per capita across countries, it will not be enough to provide any information on the variations of socioeconomic resources that may drive these CVD outcomes. Potential effect measure modifiers at the individual level (eg, age, sex, and education) were not examined. However, in terms of internal validity, we note that our results were robust to multiple adjustments of potential time-varying confounders. On the other hand, exposure misclassification may result from assigning measured ambient temperature averaged from monitoring stations to all individuals at a study site. Exposure misclassification will be lower for variables that have less within-city heterogeneity compared to other variables with more subscale variance (eg, temperature compared to particulate matter with aerodynamic diameter <2.5 µm). Additionally, causes of CVD deaths may be coded differently across sites leading to misclassification bias. We could not examine subtypes of heart failure such as those with preserved or reduced ejection fraction nor disentangle types of arrhythmias. Using mortality from arrhythmia as an outcome may not be the best approach, since it may be difficult to pinpoint arrhythmic death as the underlying cause. Alternatively, studying the associations between extreme temperatures and the incidence of nonfatal cardiovascular diseases (including arrhythmias) may provide us with more answers.

### Conclusions

This large, multicountry, multicity investigation shows increased risk and burden of all-cause CVD, ischemic heart disease, stroke, and heart failure mortality from extreme hot and cold temperatures. The results illustrate the relevance of environmental exposures to specific CVD causes of mortality and have direct relevance to prompt optimal medical and public health responses to temperature extremes in present day and under a changing climate.

## Article Information

### Acknowledgments

B.Alahmad, A.Z., and AG set up the collaborative network. B.Alahmad and H. Khraishah conceptualized the idea. B.Alahmad, A.V.C., and A.G. designed the study. B.Alahmad, J.S., D.R., A.V.C., and A.G. developed the statistical methods. B.Alahmad and H.Khraishah drafted the manuscript and interpreted the results. A.Z., A.V.C., D.R., Y.G., S.I.P., S.A., F.A., B.Armstrong, M.B., B.Y.C., T.N.D., D.V.D., A.E., Y.L.L.G., M.H., Y.H., E.I., C.I., J.J.K., J.S., H.Kim, E.L., W.L., S.L., J.M., F.M., H.O., A.O., M.S.R., N.R., P.H.S., N.S., X.S., F.S., S.P.S., F.D., M.S., and A.T. provided the data, and contributed to the interpretation of the results and reviewed the manuscript. P.K., J.S., A.S.B., E.G., and A.G. supervised the research project and contributed to the interpretation of the results. B.Alahmad and F.S. accessed and verified the data. All authors had the final responsibility for the decision to submit for publication.

### Sources of Funding

This study was supported by the Kuwait Foundation for the Advancement of Science (CB21-63BO-01); the US Environmental Protection Agency (RD-835872); Harvard Chan National Institute of Environmental Health Sciences Center for Environmental Health (P01ES009825); the UK Medical Research Council (MR/R013349/1); the UK Natural Environment Research Council (NE/R009384/1); the European Union’s Horizon 2020 Project Exhaustion (820655); the Australian National Health and Medical Research Council (APP 2000581, APP 1109193, APP 1163693); the National Institute of Environmental Health Sciences–funded HERCULES Center (P30ES019776); the MCIN/AEI/10.13039/501100011033 (grant CEX2018-000794-S); the Taiwanese Ministry of Science and Technology (MOST 109–2621-M-002–021); the Environmental Restoration and Conservation Agency, Environment Research and Technology Development Fund (JPMEERF15S11412); the São Paulo Research Foundation; and Fundação para a Ciência e a Tecnlogia (SFRH/BPD/115112/2016). The funders had no role in the design and conduct of the study; collection, management, analysis, and interpretation of the data; preparation, review, or approval of the manuscript; and decision to submit the manuscript for publication. The contents are solely the responsibility of the grantees and do not necessarily represent the official views of the funding agencies. Furthermore, the funding agencies do not endorse the purchase of any commercial products or services related to this publication.

### Disclosures

None.

### Supplemental Material

Supplemental Methods

Tables S1 and S2

Figures S1–S5

References 44–51

## Supplementary Material
