# Supplementary material for: Associations Between Extreme Temperatures and Cardiovascular Cause-Specific Mortality: Results From 27 Countries
Source: Circulation. 2022 Dec 12;147(1):35–46. doi: 10.1161/CIRCULATIONAHA.122.061832 (PMC9794133; doi:10.1161/CIRCULATIONAHA.122.061832)

SUPPLEMENTAL MATERIAL

Cardiovascular Cause-specific Mortality and Extreme Temperatures: Results from 27 Countries – *Alahmad et al.*

Table of Content

Detailed Methods. Additional information on the statistical analysis

Table S1. Description of the temperature and cardiovascular-specific mortality data sources in the MCC locations

Table S2. Temperature percentiles and Minimum Mortality Temperature (MMT) for all-cause cardiovascular deaths across all countries

Table S3. Descriptive statistics for each location included in the study

Table S4. Relative risk of death for extreme cold (1st vs. MMT) and extreme heat (99th vs. MMT) across all countries

Table S5. Excess deaths (per 1000 deaths) for a range of extreme cold temperatures (2.5th and below) and a range of extreme hot temperatures (97.5th and above)

Table S6. Excess deaths (per 1000 deaths) for all cold temperatures (below the MMT) and all hot temperatures (above the MMT)

Table S7. Top 5 cities with the highest relative risk of cause-specific CVD death, stratified by quartiles of GDP per capita

Table S8. Heterogeneity parameters in meta regression models

Table S9. Sensitivity analyses and effect estimates for all-cause cardiovascular mortality

Figure S1. Distribution of ambient temperatures in all 27 countries. Minimum mortality temperatures (for all-cause cardiovascular mortality) are shown in vertical dotted lines.

Figure S2. Pooled exposure-response relationships between temperature (in the absolute scale; °C) and relative risk (RR) of different causes of CVD mortality

Figure S3**.** Exposure-response relationships between temperature (in the absolute scale; °C) and relative risk (RR) of all-cause CVD mortality in selected 12 cities around the world.

Figure S3. Pooled exposure-response relationships between temperature percentiles and relative risk (RR) of different causes of CVD mortality with stratification at the 25th percentile and 75th percentile of country-level gross domestic product (GDP) per capita.

Figure S4. Pooled exposure-response relationships between temperature percentiles and relative risk (RR) of different causes of CVD mortality with stratification at the 25th percentile and 75th percentile of city-specific mean winter temperature.

Figure S5. Pooled exposure-response relationships between temperature percentiles and relative risk (RR) of different causes of CVD mortality with stratification at the 25th percentile and 75th percentile of city-specific mean summer temperature.

Detailed Methods: Additional information on the statistical analysis

Overarching statistical approach:

1) ***Stage 1:*** lagged and non-linear temperature-CVD relationships at each city
2) ***Stage 2:*** a hierarchical pooling approach (mixed effects meta-analysis)
3) ***Burden measures:*** estimating excess deaths from CVD causes

The analysis was repeated for each CVD outcome separately (all-cause cardiovascular, ischemic heart disease, stroke, heart failure and arrhythmia). All analyses were carried out using R software (version 3.6.0) with *gnm* [44], *dlnm* [45] and *mixmeta* [19] packages.

Stage 1 – In a case-crossover study design, each individual case serves as their own control. This approach is commonly used in environmental epidemiology to estimate the short-term relationship between acute health events and environmental exposures, effectively eliminating any possible confounding from individual characteristics such as age, gender, diet, smoking and so on. [16] However, since we did not have individual-level data, we used counts of deaths in conditional Poisson models as a flexible time series alternative to the conventional case-crossover conditional logistic regression.[17] More specifically:

- We fitted conditional quasi-Poisson models for each location by including a three-way interaction terms between year, month and day of the week. [17]
- This is done using generalized non-linear models (*gnm*) in which a stratum (e.g., Wednesdays of March in 2018) that has no deaths is eliminated from the model as it does not contribute information and could artificially impact the confidence intervals of the estimated coefficients.
- The indicator variables from the interaction terms are not estimated, rather they are 'conditioned out' by conditioning on the sum of events in each stratum.
- The quasi-likelihood approach handles the potential risk of over- or under-dispersion.
- The temperature-lag-mortality association in each location is modelled with distributed lag non-linear models (*DLNM*).
- Using this method, we can capture the complex non-linear exposure-response relationship as well as the additional lagged dependencies.
- The bi-dimensional *DLNM* function of temperature and lag allows for a simultaneous estimation of different non-linear associations of temperature at each lag and across lags.
- The lag-response association represents the temporal change in risk after a specific exposure, and it estimates the distribution of immediate and delayed effects that cumulate across the lag period.
- Smoothing for temperature is independent from lag smoothing; temperature was smoothed with a quadratic B-spline with three internal knots placed at the 10th, 75th, and 90th percentiles of each location while the lag was modeled with natural splines with three internal knots equally spaced in the log scale, consistent with previous MCC studies. [5,18]
- The lag period was extended to only 14 days to account for long delay and 'harvesting' (a phenomenon where deaths are occurring only a few days early among persons who were already dying).
- For each location and cause of death, we computed the overall cumulative relative risk (RR) corresponding to each day's temperature compared to the minimum mortality temperature (MMT) which is the temperature that is associated with the least mortality risk.
- The MMTs are empirically estimated without imposing constraints on its location.

- There is evidence that suggest that the MMT is a potential indicator of human long-term adaptation to heat. [21,46]

Stage 2 – At this stage we perform a hierarchical pooling approach using a novel mixed effects meta-analysis that allows fitting fixed and random effects for the temperature-CVD mortality relationships across all cities. More specifically:

- Parameters from ***Stage 1*** are reduced to only the cumulative risk during the lag period for each location. This step allows us to have fewer parameters for pooling without compromising the complexity of the delayed non-linear estimates. [47]
- We then applied a novel multilevel meta-analysis that allows complex random effects for nested groups (*mixmeta*), as described by Sera et al. (2019). [19]
- A linear mixed effects (LME) model that can be written as:

$$\boldsymbol{y_i} = \boldsymbol{X_i\beta} + \boldsymbol{Z_i b_i} + \boldsymbol{\epsilon_i}$$

$$\boldsymbol{b_i} \sim \boldsymbol{N}(\boldsymbol{0}, \boldsymbol{\Psi_i}) \qquad \boldsymbol{\epsilon_i} \sim \boldsymbol{N}(\boldsymbol{0}, \boldsymbol{S_i})$$

  Where $\mathbf{y}_i$ are effect sizes (outcomes) in group i, $\mathbf{X}_i\boldsymbol{\beta}$ is the fixed effects that represent the population-averaged outcomes, $\mathbf{Z}_i$ is the random-effects design matrix with coefficients $\mathbf{b}_i$, vector $\boldsymbol{\epsilon}_i$ defines the unit-level sampling errors, and $\mathbf{S}_i$ and $\boldsymbol{\Psi}_i$ are (co)variance matrices that define within-group errors and between-group random effects, respectively. Random effects models were fitted using maximum likelihood estimation.
- To account for possible effect measure modification on the pooled relationship, we used the following variables as fixed meta-predictors in the meta-regression:
  - location-specific mean summer temperature: average temperature for the range of temperatures that are above the median in each location.
  - location-specific mean winter temperature: average temperature for the range of temperatures that are below the median in each location.
  - country-level gross domestic product (GDP) per capita (in current US Dollars): we used the GDP per capita that corresponds to the last year available in the mortality series in each country from the World Bank (https://data.worldbank.org/indicator/NY.GDP.PCAP.CD).
- We also fitted a two-level random effects where cities are nested within country-specific climate zones (the Köppen-Geiger climate classifications [15]) allowing cities in the same country and climate zone to borrow information from each other.
- We predicted the pooled and country-level relative risks from the meta-regression model.
- The RR of death for each CVD outcome is reported for extreme cold as the location-specific 1st percentile temperature vs. the MMT, and extreme heat as the 99th percentile vs. the MMT.
- Heterogeneity between locations was examined using an extended form of the Cochran Q test and I^2 statistic. [20] The heterogeneity in the main model with meta predictors and random effects was compared to a pooling model with intercept only.

Burden measures **–** To understand the burden of extreme temperatures on cause-specific CVD mortality, we calculated the attributable mortality (excess deaths) from a range of extreme cold or hot temperatures at each location. More specifically:

- The Best Linear Unbiased Predictions (BLUP) were then extracted for each city.
- The MMT for each city was re-centered based on the BLUP.

- Using the BLUP, we used the overall cumulative RR (over the next 14 days) for each day of the series above and below extreme temperature thresholds to calculate number of excess deaths (attributable number). The numbers were summed across cities within the same country.
- Extreme range of cold days was defined as all days below the 2.5th location-specific temperature percentile, while extreme range of hot days was defined as all days above the 97.5th location-specific temperature percentile. These definitions are consistent with previous heatwave literature and multi-country temperature studies [18,48,49].
- In an additional analysis, we also calculated the excess deaths associated with the whole range of hot temperatures above the MMT and cold temperatures below the MMT.
- For temperature x_t in a given day t, the attributable number $AN_{x,t}$ of deaths are those experienced in the next l lag days and L as the maximum lag period, where $\sum \beta_{x_t,l}$ is the overall cumulative log-relative risk for temperature x_t and n_t is the number of deaths in day t [50]:

$$AN_{x,t} = \left(1 - exp\left(-\sum_{l=0}^{L} \beta_{x_t,l}\right)\right)\left(\sum_{l=0}^{L} \frac{n_{t+1}}{L+1}\right)$$

- We divide the excess death numbers by total deaths from each CVD cause. Proportion of excess deaths that are attributable to ranges of extreme temperature are then obtained. They are expressed as number of excess deaths for each 1,000 cause-specific deaths.
- We calculated empirical confidence intervals (eCI) using Monte Carlo simulations assuming a multivariate normal distribution of the BLUP of reduced coefficients.

Sensitivity analyses – We conducted a number of sensitivity analyses to assess the robustness of the results based on the modelling choices made in the main analysis.

Main model: Temperature: quadratic B-spline with 3 internal knots placed at the 10th, 75th, and 90th percentiles.
Lag period: 14 days
Lag modelling: natural splines with 3 internal knots spaced equally in the log scale
Adjustment to other environmental exposures: none

Alternative approach 1: Temperature: quadratic B-spline with 4 internal knots placed at the 10th, 50th, 75th, and 90th percentiles.

Alternative approach 2: Temperature: quadratic B-spline with 5 internal knots placed at the 5th, 25th, 50th, 75th, and 95th percentiles.

Alternative approach 3: Lag period: 21 days

Alternative approach 4: Adjusting for very long-term decadal changes in trends: natural splines for time with 1 degree of freedom per decade.

Alternative approach 5: Adjusting for heatwave indicator (0/1): defined as any 2 consecutive days > 95th percentile (i.e., duration of extreme events)

Alternative approach 6: Adjusting for heatwave indicator (0/1): defined as any 2 consecutive days > 99th percentile (i.e., duration of extreme events)

Alternative approach 7: Adjusting for inter-day temperature variability: defined as the absolute change in temperature between 2 neighboring days. We built two independent indicators (delta temperature increase/decrease) of inter-day temperature variation to account for hot and cold seasons when adjusted for simultaneously:

$$\Delta\, temp_i^{inc} = \max\,[temp_i^{mean} - \max(temp_{i-1}^{mean}, MMT)\,, 0]$$

And;

$$\Delta\, temp_i^{dec} = \max\,[\min(temp_{i-1}^{mean}, MMT) - temp_i^{mean}, 0]$$

This approach is described in more details in Vicedo-Cabrera et al. [51]

Alternative approach 8: Adjustment to other environmental exposures: relative humidity (24-hr average, in %)

Alternative approach 9: Adjustment to other environmental exposures: ozone (O_3) (maximum 8-hour average, in µg/m^3, data described previously [11])

Alternative approach 10: Adjustment to other environmental exposures: nitrogen dioxide (NO_2) (24-hr average, in µg/m^3, data described previously [14])

Alternative approach 11: Adjustment to other environmental exposures: particulate matter of aerodynamic diameter less than or equal 10 µm (PM_{10}) (24-hr average, in µg/m^3, data described previously [12])

Alternative approach 12: Adjustment to other environmental exposures: particulate matter of aerodynamic diameter less than or equal 2.5 µm ($PM_{2.5}$) (24-hr average, in µg/m^3, data described previously [12])

In alternative approaches 8 to 12, the effect estimates were compared to the main model that is restricted to the locations where the environmental exposure data was available.

Table S1. Description of the temperature and cardiovascular-specific mortality data sources in the MCC locations.

COUNTRY	N LOCATIONS	PERIOD	MORTALITY DATA	TEMPERATURE DATA	MISSING DATA
Brazil	12	1997-2018	Cardiovascular specific mortality collected from the Ministry of Health.	Mean daily temperature (in ˚C) computed from the 24-h average of hourly measurements, from weather stations located within the urban area provided by National Institute of Meteorology of Brazil	Missing data amount for 2.404%, 4.157%, 13.171%, 19.44%, 40.859% and 38.867% of the temperature, all-cause CVD, stroke, IHD, HF and arrhythmia series, respectively.
Canada	26	1986-2015	Cardiovascular specific mortality collected from Canadian Mortality Database.	Mean daily temperature (in ˚C) computed as the 24-hour average based on hourly measurements, were obtained from Environment Canada collected from monitoring stations located closest to the CMA centre.	Missing data amount for 0.361%, 0%, 0%, 0%, 0% and 0% of the temperature, all-cause CVD, stroke, IHD, HF and arrhythmia series, respectively.
Costa Rica	1	2000-2017	Cardiovascular specific mortality collected from the Instituto Nacional de Estadística y Censo.	Meteorological data were obtained from WMO NOAA (Surface Data Hourly Global, DS3505)	Missing data amount for 0.35%, 0%, 0%, 0%, 0% and 0% of the temperature, all-cause CVD, stroke, IHD, HF and arrhythmia series, respectively.
Cyprus	5	2004-2017	Cardiovascular specific mortality collected from Causes of Death Database, Health Monitoring Unit, Ministry of Health of the Republic of Cyprus	Mean daily temperature (in ˚C), computed as the average between daily minimum and maximum, were obtained from the Department of Meteorology of Cyprus, Ministry of Agriculture, Rural Development and Environment	Missing data amount for 0%, 0%, 0%, 0%, 0% and 0% of the temperature, all-cause CVD, stroke, IHD, HF and arrhythmia series, respectively.
Ecuador	2	2013-2019	Cardiovascular specific mortality collected from the Instituto Nacional de Estadística y Censos.	Meteorological data were obtained from WMO NOAA (Surface Data Hourly Global, DS3505)	Missing data amount for 4.264%, 0%, 0%, 0%, 0% and 0% of the temperature, all-cause CVD, stroke, IHD, HF and arrhythmia series, respectively.
Estonia	9	1997-2018	Cardiovascular specific mortality collected from *Estonian Causes of Death Registry*	Mean daily temperature (in ˚C) were computed as the 24-h average of hourly measurements collected from *Estonian Environment Agency*.	Missing data amount for 0%, 0%, 0%, 0%, 0% and 0% of the temperature, all-cause CVD, stroke, IHD, HF and arrhythmia series, respectively.
Finland	1	1987-2018	Cardiovascular specific mortality collected from Statistics Finland	Mean daily temperature (in ˚C), Finnish Meteorological Institute. The weather stations around the country were interpolated onto a 10×10 km grid covering the whole of Finland, using a Kriging model.	Missing data amount for 0%, 0%, 0%, 0%, 0% and 0% of the temperature, all-cause CVD, stroke, IHD, HF and arrhythmia series, respectively.
Guatemala	1	2009-2018	Cardiovascular specific mortality collected from the Instituto Nacional	Temperature data are	Missing data amount for 0.767%, 0%, 0%, 0%, 0% and 0% of the temperature, all-cause

			de Estadística, Unidad de Estadística de Salud.	provided by the Instituto Nacional de Sismología, Vulancología, Meteorología y Hidrología.	CVD, stroke, IHD, HF and arrhythmia series, respectively.
Iran	2	2001-2017	Cardiovascular specific mortality collected from Ferdows Organization of Mashhad Municipality and Behesht Zahra Organization of Tehran Municipality	Temperature data are provided by Iran Meteorological Organization	Missing data amount for 7.558%, 0%, 0%, 0%, 0% and 0% of the temperature, all-cause CVD, stroke, IHD, HF and arrhythmia series, respectively.
Italy	6	2006-2015	Cardiovascular specific mortality collected from regional mortality registry of the Lazio Region	Mean daily temperature (in ˚C) was computed as the 24-h average based on 6-h measurements obtained from the Meteorological Service of the Italian Air Force. A single weather station was selected for each city, using the airport monitoring station located closest to the city center.	Missing data amount for 1.543%, 0%, 0%, 0%, 0% and 0% of the temperature, all-cause CVD, stroke, IHD, HF and arrhythmia series, respectively.
Japan	47	1979-2015	Cardiovascular specific mortality collected from Ministry of Health, Labour and Welfare.	Weather station located within the urban area of the capital city (Japan Meteorology Agency)	Missing data amount for 0.03%, 0%, 0%, 0%, 0% and 0% of the temperature, all-cause CVD, stroke, IHD, HF and arrhythmia series, respectively.
Kuwait	1	2000-2016	Cardiovascular specific mortality collected from the National Center for Health Information, Ministry of Health, Kuwait.	Mean daily temperature (in ˚C) computed as the 24-hour average based on hourly measurements from two sources: the Directorate General of Civil Aviation (Kuwait Airport), and Kuwait's Environmental Public Authority.	Missing data amount for 0.032%, 0%, 0%, 0%, 0% and 0% of the temperature, all-cause CVD, stroke, IHD, HF and arrhythmia series, respectively.
Moldova	1	2001-2010	Cardiovascular specific mortality provided by National Centre for Health Management	Mean daily temperature (in ˚C) computed as the average between daily minimum and maximum, were obtained from State Hydrometeorological Service, Moldova.	Missing data amount for 0%, 0%, 0%, 0%, 100% and 100% of the temperature, all-cause CVD, stroke, IHD, HF and arrhythmia series, respectively.
Panama	1	2013-2016	Cardiovascular specific mortality provided by Instituto Nacional de Estadística y Censo, Centro de Información Estadística.	Temperature data are provided by the Empresa de Transmisión Eléctrica, S.A. (ETESA). Open Access.	Missing data amount for 8.487%, 0%, 0%, 0%, 0% and 0% of the temperature, all-cause CVD, stroke, IHD, HF and arrhythmia series, respectively.
Paraguay	1	2004-2019	Cardiovascular specific mortality provided by Ministerio de Salud Pública y Bienestar	Temperature data are obtained from the Global Historical Climatology Network (NOAA/WMO)	Missing data amount for 0.53%, 0%, 0%, 0%, 0% and 0% of the temperature, all-cause CVD, stroke, IHD, HF and arrhythmia series, respectively.

			Social, Dirección General de Información Estratégica en Salud, Subsistema de Información de Estadísticas Vitales		
Philippines	4	2006-2010	Cardiovascular specific mortality provided by Philippine Statistics Agency	Mean daily temperature (in ˚C), computed as 24-hour average based on hourly measurements, were obtained from National Oceanic and Atmospheric Administration (NOAA)	Missing data amount for 0.014%, 0%, 0%, 0%, 0% and 0% of the temperature, all-cause CVD, stroke, IHD, HF and arrhythmia series, respectively.
Portugal	6	1990-2018	Cardiovascular specific mortality provided by Statistics Portugal.	Mean daily temperature (in ˚C) was computed as the 24-hour average based on hourly measurements collected from the National Oceanic and Atmospheric Administration (NOAA)	Missing data amount for 2.419%, 0%, 0%, 0%, 0% and 0% of the temperature, all-cause CVD, stroke, IHD, HF and arrhythmia series, respectively.
South Africa	52	1997-2013	Cardiovascular specific mortality provided by Statistics South Africa	Mean daily temperature (in ˚C) was computed as the average between daily minimum and maximum collected from the Agricultural Research Council of South Africa and the National Oceanic and Atmospheric Administration (NOAA).	Missing data amount for 6.271%, 0%, 0%, 0%, 0% and 0% of the temperature, all-cause CVD, stroke, IHD, HF and arrhythmia series, respectively.
South Korea	36	1997-2018	Cardiovascular specific mortality collected from provided by Korea Bureau of Statistics	Mean daily temperature (in ˚C) computed as the 24-hour average based on hourly measurements, were obtained from weather stations located within the urban area managed by Korea Meteorological Administration.	Missing data amount for 0.014%, 0%, 0%, 0%, 100% and 100% of the temperature, all-cause CVD, stroke, IHD, HF and arrhythmia series, respectively.
Spain	6	2000-2018	Cardiovascular specific mortality collected from the Spain National Institute of Statistics.	Mean daily temperature (in ˚C), computed as the 24-hour average based on hourly measurements, and was obtained from weather stations of the Spain National Meteorology Agency. A single weather station, located within the urban area or at the near airport, was selected for each city	Missing data amount for 0.007%, 0%, 0%, 0%, 0% and 0% of the temperature, all-cause CVD, stroke, IHD, HF and arrhythmia series, respectively.
Switzerland	8	1995-2016	Cardiovascular specific mortality provided from	Mean daily temperature (in ˚C) computed as the 24-hour average based on hourly measurements, were	Missing data amount for 0%, 0%, 0%, 0%, 0% and 0% of the temperature, all-cause

			Federal Office of Statistics (Switzerland)	obtained from the IDAWEB database (a service provided by MeteoSwiss, the Swiss Federal Office of Meteorology and Climatology). A single weather station located within or near the urban area was selected for each city.	CVD, stroke, IHD, HF and arrhythmia series, respectively.
Taiwan	3	2008-2016	Cardiovascular specific mortality provided by the Department of Health in Taiwan	Mean daily temperature (in ˚C) were computed as the 24-hour average based on hourly measurements provided by Taiwan Environmental Protection Agency	Missing data amount for 0.02%, 0%, 0%, 0%, 0% and 0% of the temperature, all-cause CVD, stroke, IHD, HF and arrhythmia series, respectively.
Thailand	55	1999-2008	Cardiovascular specific mortality provided the Ministry of Public Health, Thailand.	Mean daily temperature (in ˚C) computed as the average between daily minimum and maximum, were obtained from the Meteorological Department, Ministry of Information and Communication Technology, Thailand.	Missing data amount for 2.797%, 0%, 0%, 0%, 0% and 0% of the temperature, all-cause CVD, stroke, IHD, HF and arrhythmia series, respectively.
UK	70	1990-2016	Cardiovascular specific mortality provided by the Office of National Statistics.	Mean daily temperature (in ˚C) were computed as the 24-hour average based on hourly measurements from UKCP09 5kmx5km product	Missing data amount for 0%, 0%, 0%, 0%, 0% and 0% of the temperature, all-cause CVD, stroke, IHD, HF and arrhythmia series, respectively.
Uruguay	1	2001-2018	Cardiovascular specific mortality provided by the Ministerio de Salud Publica (MSP).	Temperature data are provided by the Instituto Uruguayo de Meteorología (INUMET)	Missing data amount for 0.109%, 0%, 0%, 0%, 0% and 0% of the temperature, all-cause CVD, stroke, IHD, HF and arrhythmia series, respectively.
USA	209	1985-2006	Cardiovascular specific mortality provided by the National Center for Health Statistics (NCHS).	Mean daily temperature (in ˚C) computed as the 24-hour average based on hourly measurements, were obtained from the National Climatic Data Center (NCDC) of the National Oceanic and Atmospheric Administration (NOAA).	Missing data amount for 0.12%, 0%, 0%, 0%, 0% and 0% of the temperature, all-cause CVD, stroke, IHD, HF and arrhythmia series, respectively.
Vietnam	1	2010-2013	Cardiovascular specific mortality provided by Provincial Department of Health.	Mean daily temperature (in ˚C), computed as computed from the 24-h average of hourly measurements, were obtained from National Oceanic and Atmospheric Administration's (NOAA) National Climate Data Center (NCDC).	Missing data amount for 0%, 0%, 0%, 0%, 0% and 0% of the temperature, all-cause CVD, stroke, IHD, HF and arrhythmia series, respectively.

Table S2. Temperature percentiles and minimum mortality temperature (MMT) for all-cause cardiovascular deaths across all countries

	Country	1st (°C)	2.5th (°C)	MMT Percentile (%)	MMT (°C)	97.5th (°C)	99th (°C)
**North America**							
**1**	Canada	-13.09	-11.20	92	19.60	21.05	22.01
**2**	US	-2.45	-0.84	90	24.27	25.86	26.6
**Caribbean and Central America**							
**3**	Guatemala	14.29	15.55	87	21.00	21.95	22.42
**4**	Costa Rica	20.20	20.66	84	23.83	25.00	25.36
**5**	Panama	25.24	25.80	79	29.10	30.30	30.60
**South America**							
**6**	Uruguay	7.80	8.87	85	25.15	28.74	30.11
**7**	Ecuador	18.68	18.99	88	22.35	26.21	27.36
**8**	Paraguay	9.68	11.51	82	28.22	31.24	32.17
**9**	Brazil	19.28	19.99	81	25.76	27.29	27.68
**South Africa**							
**10**	South Africa	9.14	10.01	86	22.66	24.08	24.6
**North Europe**							
**11**	Finland	-17.2	-12.69	93	18.40	20.9	22.4
**12**	Estonia	-15.77	-11.69	93	18.37	20.98	22.56
**13**	UK	-0.55	0.88	92	17.56	19.59	20.76
**Central Europe**							
**14**	Switzerland	-4.17	-2.51	91	20.45	23.12	24.58
**15**	Moldova	-11.05	-8.40	92	23.90	26.50	28.2
**South Europe**							
**16**	Portugal	6.61	7.66	89	22.76	25.87	27.49
**17**	Spain	6.19	7.22	86	24.70	27.03	27.75
**18**	Italy	3.14	4.48	88	25.87	28.87	29.65
**19**	Cyprus	8.30	10.03	81	27.60	29.86	30.7
**Middle–East Asia**							
**20**	Iran	-2.15	-0.40	91	27.60	31.00	32.88
**21**	Kuwait	9.11	10.34	79	37.33	41.12	42.08
**East Asia**							
**22**	South Korea	-5.44	-3.63	91	25.26	27.5	28.29
**23**	Japan	1.19	2.14	90	26.32	28.3	28.87
**24**	Taiwan	12.90	14.04	78	28.66	30.56	31.00
**South East Asia**							
**25**	Thailand	22.25	23.19	81	29.01	30.68	31.27
**26**	Philippines	25.55	26.09	82	29.15	30.43	30.74
**27**	Vietnam	25.28	25.70	81	29.68	31.00	31.58

Table S3. Descriptive statistics for each location included in the study

Location	Country	Years	Temperature mean (SD) °C	Climate Zone	All CVD	IHD	Stroke	HF	Arrhythmia
Abbotsford	Canada	1986-2015	10.63 (6.17)	C	16378	8453	3502	1067	624
Calgary	Canada	1986-2015	4.72 (10.32)	D	55482	29474	10183	2274	1074
Edmonton	Canada	1986-2015	3.28 (11.79)	D	63429	34900	12212	2819	1312
Halifax	Canada	1986-2015	7.06 (9.46)	D	26707	13505	4900	1825	804
Hamilton	Canada	1986-2015	8.37 (10.32)	D	42389	24749	7934	1890	972
Kingston	Canada	1986-2015	7.67 (10.76)	D	17632	9817	3629	776	368
Kitchener-Waterloo	Canada	1986-2015	7.27 (10.52)	D	28677	16165	5959	993	803
London Ontario	Canada	1986-2015	8.12 (10.47)	D	34623	19078	6882	1635	866
Montreal	Canada	1986-2015	7.21 (11.76)	D	149812	85449	26013	9452	4254
Niagara	Canada	1986-2015	9.42 (10.15)	D	43913	27231	8198	1437	774
Oakville	Canada	1986-2015	8.87 (10.07)	D	22632	12851	4687	962	687
Oshawa	Canada	1986-2015	8.29 (10)	D	28276	15987	5882	1203	666
Ottawa	Canada	1986-2015	6.64 (11.93)	D	61250	34357	11222	2678	1468
Regina	Canada	1986-2015	3.03 (13.41)	D	20313	10624	3868	1411	376
Saint John NB	Canada	1986-2015	5.35 (9.83)	D	18619	10075	3454	1525	350
Sarnia	Canada	1986-2015	8.63 (10.23)	D	13244	7770	2665	431	337
Saskatoon	Canada	1986-2015	2.67 (13.56)	D	23411	11607	4647	1548	661
Sault Ste. Marie	Canada	1986-2015	5.01 (11.01)	D	11753	7145	2144	514	202
St. John's NFL	Canada	1986-2015	5.48 (8.23)	D	23738	13200	4643	1250	382
Sudbury	Canada	1986-2015	4.29 (12.31)	D	16834	10117	2802	637	275
Thunder Bay	Canada	1986-2015	3.1 (12.1)	D	14936	8935	2819	501	353
Toronto	Canada	1986-2015	8.76 (10.15)	D	263173	144056	55614	12430	6527
Vancouver	Canada	1986-2015	10.84 (5.63)	C	130383	65945	30040	8035	3856
Victoria	Canada	1986-2015	10.54 (4.95)	C	34632	15799	7965	2506	1235
Windsor	Canada	1986-2015	10.05 (10.51)	D	33513	18677	6406	1150	507
Winnipeg	Canada	1986-2015	3.48 (14.08)	D	68860	36689	14254	3981	1562
Akron, OH	US	1985-2006	10.2 (10.07)	D	43535	20970	6986	3515	826

Albany, NY	US	1985-2006	9.28 (10.38)	D	28238	16755	3736	1396	481
Albuquerque, NM	US	1985-2006	14.09 (8.94)	B	26813	11472	4923	1625	457
Allentown, PA	US	1985-2006	11.21 (9.73)	C	48711	24739	6636	2488	1049
Anaheim, CA	US	1985-2006	18.85 (4.42)	B	143296	83450	25168	3817	457
Anchorage, AK	US	1985-2006	3.26 (9.96)	C	5707	2668	1162	238	113
Ann Arbor, MI	US	1985-2006	9.65 (10.36)	D	13709	7068	2446	771	271
Annandale, VA	US	1985-2006	14.15 (9.37)	C	26326	10378	4922	2099	652
Atlanta, GA	US	1985-2006	16.32 (8.07)	C	119625	45527	21347	7223	2689
Atlantic City, NJ	US	1985-2006	12.37 (9.21)	C	21330	12029	2994	654	245
Augusta, GA	US	1985-2006	17.89 (7.83)	C	15040	7178	2514	677	276
Austin, TX	US	1985-2006	20.35 (7.63)	C	26311	11718	5196	1317	540
Aztec, NM	US	1985-2006	11.43 (9.72)	B	2574	1030	391	182	NA
Bakersfield, CA	US	1985-2006	18.36 (7.85)	B	39358	23798	5738	1292	296
Baltimore, MD	US	1985-2006	13.69 (9.45)	C	128316	55378	20201	5516	2650
Bangor, ME	US	1985-2006	7.22 (10.59)	D	11029	5406	1831	658	189
Barnstable, MA	US	1985-2006	10.6 (8.64)	C	20452	9133	3803	1613	425
Bath, NY	US	1985-2006	9.02 (9.97)	D	5956	3310	881	350	105
Baton Rouge, LA	US	1985-2006	19.66 (7.19)	C	26460	11332	3537	2248	319
Beaver Dam, WI	US	1985-2006	8.52 (11.18)	D	5595	2965	1057	342	NA
Birmingham, AL	US	1985-2006	17.12 (8.22)	C	69157	26912	13160	6172	2095
Boise City, ID	US	1985-2006	11.22 (9.87)	C	8713	4729	1546	406	152
Boston, MA	US	1985-2006	10.77 (9.33)	D	186695	96398	30134	12917	4481
Boulder, CO	US	1985-2006	11.94 (10.02)	B	9545	3674	1978	634	162
Brownsville, TX	US	1985-2006	23.14 (5.74)	C	14026	7495	2398	593	146
Buffalo, NY	US	1985-2006	9.08 (9.92)	D	99052	58107	15548	3874	1253
Burlington, VT	US	1985-2006	7.97 (11.1)	D	7015	3351	1080	311	141
Canton, OH	US	1985-2006	10.08 (10.08)	C	33737	18068	5575	1626	632
Carlisle, PA	US	1985-2006	12.38 (9.81)	C	17192	9211	2930	1068	313
Cedar Rapids, IA	US	1985-2006	9.37 (11.45)	D	11337	5612	2105	295	167
Charleston, SC	US	1985-2006	18.71 (7.35)	C	20087	9432	4225	990	420
Charleston, WV	US	1985-2006	12.99 (9.15)	C	20681	9751	2993	1227	351

Charlotte, NC	US	1985-2006	16.12 (8.27)	C	31840	14478	6394	1506	596
Chattanooga, TN	US	1985-2006	15.82 (8.54)	C	27061	12420	4599	780	359
Chicago, IL	US	1985-2006	11.31 (10.57)	D	481463	255004	72530	21706	9291
Cincinnati, OH	US	1985-2006	12.69 (9.65)	C	71685	34403	11577	3477	1092
Cleveland, OH	US	1985-2006	11.22 (10.09)	D	181755	109137	24627	8521	2573
Colorado Springs, CO	US	1985-2006	9.44 (9.34)	B	19047	8929	4704	676	231
Columbia, SC	US	1985-2006	17.62 (8.11)	C	29969	15346	5812	1505	566
Columbus, OH	US	1985-2006	11.79 (10.06)	C	63129	29312	10199	3268	1380
Corpus Christi, TX	US	1985-2006	22.05 (6.37)	C	18229	8779	3276	882	246
Dallas, TX	US	1985-2006	19.54 (8.86)	C	109906	49584	19232	4453	1214
Davenport, IA	US	1985-2006	10.2 (11.21)	D	24901	13235	4636	1581	322
Dayton, OH	US	1985-2006	11.64 (10.13)	C	45624	22424	7496	1896	750
Daytona Beach, FL	US	1985-2006	21.45 (5.45)	C	47542	25728	7675	1052	622
Denver, CO	US	1985-2006	10.42 (9.75)	B	69936	33227	11248	3619	1260
Des Moines, IA	US	1985-2006	10.38 (11.52)	D	23023	9973	3473	694	233
Detroit, MI	US	1985-2006	10.57 (10.44)	D	337954	182383	47338	14506	4903
Dover, DE	US	1985-2006	13.19 (9.25)	C	8551	4661	1241	406	191
Durham, NC	US	1985-2006	15.5 (8.48)	C	12303	5714	2413	769	326
East St. Louis, IL	US	1985-2006	14.51 (10.5)	C	20830	10702	3593	1413	341
El Centro, CA	US	1985-2006	23.3 (8.23)	B	6646	3560	1282	223	NA
El Paso, TX	US	1985-2006	18.35 (8.5)	B	27738	13223	4743	1117	388
Elizabeth, NJ	US	1985-2006	13.01 (9.68)	C	41734	23559	5986	1695	665
Elkhart, IN	US	1985-2006	11.63 (10.85)	D	12109	6047	2262	754	157
Erie, PA	US	1985-2006	10.13 (9.91)	D	24519	12760	4470	1290	434
Essex, MA	US	1985-2006	8.18 (10.85)	C	55711	29272	8638	3556	1233
Eugene, OR	US	1985-2006	11.45 (6.09)	C	20140	8993	4483	1380	524
Evansville, IN	US	1985-2006	13.71 (9.93)	C	16280	7895	2911	1227	432
Everett, WA	US	1985-2006	11.41 (5.75)	C	27465	13104	5485	1081	360
Fargo, ND	US	1985-2006	5.95 (13.32)	D	5820	2886	1177	356	124
Fayetteville, NC	US	1985-2006	17.05 (8.44)	C	14173	7651	2327	538	212
Flint, MI	US	1985-2006	9.04 (10.47)	D	34700	18878	5621	2170	481

Fort Lauderdale, FL	US	1985-2006	24.92 (3.93)	A	141042	87582	20412	2974	1277
Fort Myers, FL	US	1985-2006	23.4 (4.41)	A	39229	21691	5319	654	356
Fort Pierce, FL	US	1985-2006	22.92 (4.63)	C	29360	16849	4323	456	309
Fort Wayne, IN	US	1985-2006	10.37 (10.47)	D	22346	11598	3965	1745	370
Fort Worth, TX	US	1985-2006	18.91 (8.94)	C	74647	36955	13524	3169	1046
Fresno, CA	US	1985-2006	17.81 (7.87)	B	45092	24359	8145	2094	524
Gainesville, FL	US	1985-2006	20.14 (6.13)	C	9800	4696	2186	332	154
Gary, IN	US	1985-2006	11.65 (10.61)	D	39922	21587	6425	2533	861
Gettysburg, PA	US	1985-2006	12.74 (9.94)	C	5024	2619	686	314	120
Grand Haven, MI	US	1985-2006	9.14 (9.93)	D	11339	5412	2045	1025	220
Grand Junction, CO	US	1985-2006	11.82 (10.56)	B	5292	2891	865	261	104
Grand Rapids, MI	US	1985-2006	9.16 (10.42)	D	33901	14691	5607	1752	481
Green Bay, WI	US	1985-2006	7.55 (11.19)	D	13668	6714	2688	1169	227
Greensboro, NC	US	1985-2006	14.77 (8.56)	C	27119	13447	5829	1079	508
Greensburg, PA	US	1985-2006	12.42 (10.05)	C	40559	23031	5671	2108	786
Greenville, SC	US	1985-2006	17.19 (8.33)	C	24094	11721	4314	1209	565
Harrisburg, PA	US	1985-2006	12.38 (9.81)	C	22257	10458	3188	1387	1001
Hartford, CT	US	1985-2006	11.3 (9.75)	D	67519	33517	9882	3144	1304
Hickory, NC	US	1985-2006	14.96 (8.31)	C	9486	4761	1852	527	184
Holland, MI	US	1985-2006	9.23 (9.99)	D	4426	2253	694	365	NA
Honolulu, HI	US	1985-2006	25.06 (1.99)	A	33180	13528	6573	982	446
Houston, TX	US	1985-2006	20.99 (6.79)	C	150869	72284	27365	7933	1892
Indianapolis, IN	US	1985-2006	11.82 (10.33)	C	60551	30745	9768	4546	1104
Iowa City, IA	US	1985-2006	9.78 (11.44)	D	2684	1474	548	83	NA
Jacksonville, FL	US	1985-2006	21.2 (6.31)	C	50843	24975	8641	1168	706
Jersey City, NJ	US	1985-2006	10.16 (8.63)	C	43172	24805	5518	1997	798
Kalamazoo, MI	US	1985-2006	10.4 (10.72)	D	14662	7621	2591	1197	321
Kansas City, KS	US	1985-2006	13.8 (10.76)	C	90374	43309	15009	5945	2618
Kenosha, WI	US	1985-2006	9.74 (10.52)	D	10323	5277	1810	634	266
Klamath Falls, OR	US	1985-2006	8.61 (8.23)	C	3116	1581	621	153	NA
Knoxville, TN	US	1985-2006	14.86 (8.74)	C	31747	15975	6070	1047	468

La Porte, IN	US	1985-2006	10.14 (10.51)	D	9012	4730	1368	438	539
Lafayette, IN	US	1985-2006	11.36 (10.49)	C	8326	4438	1583	429	271
Lafayette, LA	US	1985-2006	19.99 (7.08)	C	9637	3991	1685	682	163
Lake Charles, LA	US	1985-2006	21.16 (7.35)	C	13689	5422	1905	886	204
Lakeland, FL	US	1985-2006	23.58 (5.17)	C	42547	24217	6085	786	421
Lancaster, PA	US	1985-2006	12.23 (9.79)	C	35667	16983	6067	2584	874
Lansing, MI	US	1985-2006	8.91 (10.46)	D	16030	7456	2748	1499	376
Las Vegas, NV	US	1985-2006	20.5 (9.64)	B	71525	29404	10585	4654	1241
Layton, UT	US	1985-2006	10.62 (10.34)	D	7049	2994	1381	582	135
Little Rock, AR	US	1985-2006	16.9 (8.99)	C	25773	11513	5729	1837	549
Logan, UT	US	1985-2006	9.03 (10.95)	D	1977	819	444	166	NA
Los Angeles, CA	US	1985-2006	17.8 (3.52)	C	577775	314393	92623	14044	1321
Louisville, KY	US	1985-2006	14.27 (9.79)	C	58088	26392	9292	4319	1265
Macon, GA	US	1985-2006	17.9 (7.77)	C	14644	6335	2718	827	249
Madison, IL	US	1985-2006	14.53 (10.51)	C	21888	12928	3367	1866	288
Madison, WI	US	1985-2006	8.6 (11.24)	D	20224	9955	4207	1333	699
Mcallen, TX	US	1985-2006	23.63 (6.17)	B	20241	12268	2979	873	203
Medford, OR	US	1985-2006	12.66 (7.68)	C	13358	6369	3180	833	255
Melbourne, FL	US	1985-2006	22.76 (4.9)	C	37140	20748	5728	755	484
Melville, NY	US	1985-2006	12.21 (9.28)	C	217729	144895	23289	7673	2069
Memphis, TN	US	1985-2006	17.18 (9.16)	C	67624	30849	12884	1928	815
Mercer, PA	US	1985-2006	9.43 (10.08)	C	12493	7377	1771	550	204
Miami, FL	US	1985-2006	24.74 (3.81)	A	167263	102258	21898	2978	1273
Middlesex, NJ	US	1985-2006	12.55 (9.78)	C	46204	27775	5828	1908	833
Middletown, OH	US	1985-2006	12.42 (9.8)	C	19960	10326	3257	1088	433
Milwaukee, WI	US	1985-2006	9.16 (10.62)	D	101013	54182	17745	4992	2466
Minneapolis, MN	US	1985-2006	8.12 (12.32)	D	88816	39902	19441	6262	1882
Mobile, AL	US	1985-2006	20.25 (7.14)	C	31072	12928	4921	1882	747
Modesto, CA	US	1985-2006	17.97 (7.57)	C	27715	15471	4790	1028	225
Monroe, LA	US	1985-2006	18.42 (8.14)	C	10825	4101	1709	732	144
Montgomery, AL	US	1985-2006	19.64 (7.86)	C	16186	6755	3056	1219	249

Muncie, IN	US	1985-2006	11.61 (10.57)	D	10084	5437	1725	537	204
Muskegon, MI	US	1985-2006	9.14 (9.93)	D	13984	7917	2340	551	218
Myrtle Beach, SC	US	1985-2006	17.81 (7.64)	C	12584	6552	2433	568	340
Nampa, ID	US	1985-2006	10.73 (9.56)	B	2875	1277	572	171	NA
Nashua, NH	US	1985-2006	10.91 (10.44)	D	21713	10800	3437	743	309
Nashville, TN	US	1985-2006	15.54 (9.17)	C	41461	19455	7341	1352	600
New Haven, CT	US	1985-2006	9.89 (11.07)	C	66935	33069	10124	3066	1192
New London, CT	US	1985-2006	11.25 (8.81)	C	16814	8005	2887	1155	199
New Orleans, LA	US	1985-2006	21.43 (6.84)	C	72211	33443	12709	4161	1141
New York, NY	US	1985-2006	10.14 (8.64)	C	638086	458730	49144	14729	3581
Newark, NJ	US	1985-2006	13.01 (9.68)	C	88571	48463	13028	4023	1736
Newburgh, NY	US	1985-2006	10.42 (9.75)	D	22079	13706	2708	1062	325
Niles, MI	US	1985-2006	10.13 (10.35)	D	13505	6990	2592	637	253
Norfolk, VA	US	1985-2006	15.9 (8.49)	C	76385	36170	13287	4964	1573
Oakland, CA	US	1985-2006	14.11 (3.36)	C	137550	66851	27887	4481	1805
Ocala, FL	US	1985-2006	20.46 (6.06)	C	25816	14392	4692	402	284
Ogden, UT	US	1985-2006	12.01 (10.68)	C	9761	4233	1849	988	177
Oklahoma City, OK	US	1985-2006	15.87 (9.89)	C	52456	25775	9216	4339	845
Omaha, NE	US	1985-2006	10.71 (11.44)	D	29582	14207	5039	1780	531
Orlando, FL	US	1985-2006	20.28 (9.94)	C	64440	35175	9698	1735	912
Ottawa, IL	US	1985-2006	10.12 (11.01)	D	11728	7188	1874	490	167
Palm Beach, FL	US	1985-2006	24.03 (4.1)	A	106240	64944	15682	1836	897
Paterson, NJ	US	1985-2006	12.37 (9.68)	C	106169	63566	14969	4384	1617
Pensacola, FL	US	1985-2006	20.07 (6.95)	C	21222	9908	4154	379	269
Philadelphia, PA	US	1985-2006	12.8 (9.54)	C	376261	179731	61062	18639	7688
Phoenix, AZ	US	1985-2006	23.9 (8.69)	B	153965	87256	25640	4665	2100
Pittsburgh, PA	US	1985-2006	11.31 (9.87)	C	141963	76600	20781	7788	2303
Plymouth, MA	US	1985-2006	8.68 (10.21)	C	31600	16312	4505	2931	750
Port Arthur, TX	US	1985-2006	20.34 (6.92)	C	22581	10401	3963	1551	295
Portage, IN	US	1985-2006	11.65 (10.61)	D	8737	4202	1393	588	400
Portland, ME	US	1985-2006	8.18 (9.7)	D	18068	8870	3045	1177	423

Portland, OR	US	1985-2006	12.26 (6.3)	C	84841	39687	18740	5068	1920
Providence, RI	US	1985-2006	10.87 (9.29)	C	112686	67335	16752	4109	1339
Provo, UT	US	1985-2006	10.66 (10.22)	D	11002	4441	2216	1286	311
Raleigh, NC	US	1985-2006	15.53 (8.49)	C	23467	11027	5079	1018	493
Reading, PA	US	1985-2006	12.23 (9.81)	C	32464	15341	5460	1559	1615
Reno, NV	US	1985-2006	11.71 (8.91)	C	18968	8478	2736	983	243
Richmond, VA	US	1985-2006	14.74 (8.94)	C	47055	19855	9086	2907	1180
Riverside, CA	US	1985-2006	17.6 (5.77)	B	193310	112129	31102	5924	1177
Rochester, NY	US	1985-2006	9.17 (10.1)	D	52751	29343	9057	3126	1300
Rockville, MD	US	1985-2006	14.37 (9.32)	C	36547	17276	6943	1585	1015
Sacramento, CA	US	1985-2006	15.79 (6.27)	C	71741	37781	13889	2391	557
Salt Lake City, UT	US	1985-2006	11.72 (10.44)	D	33312	13230	6597	3029	821
San Antonio, TX	US	1985-2006	21.26 (7.47)	C	77108	38778	13071	3279	896
San Diego, CA	US	1985-2006	17.79 (3.97)	B	155194	82180	29141	5280	1775
San Francisco, CA	US	1985-2006	14.3 (3.2)	C	102689	48833	21700	3206	900
San Jose, CA	US	1985-2006	17.29 (5.19)	C	73812	37001	14209	2394	643
Sarasota, FL	US	1985-2006	23.11 (4.95)	C	67202	37027	11522	1276	676
Scranton, PA	US	1985-2006	10.02 (9.86)	D	73933	38627	8600	3386	1421
Seattle, WA	US	1985-2006	10.76 (4.14)	C	89725	39910	18651	3723	1420
Sioux City, IA	US	1985-2006	9.51 (11.76)	D	5737	3125	926	235	NA
South Bend, IN	US	1985-2006	10.13 (10.35)	D	21585	10994	4110	1254	687
Spartanburg, SC	US	1985-2006	15.74 (8.09)	C	20162	9749	3875	1173	336
Spokane, WA	US	1985-2006	9.62 (8.74)	C	28954	13853	5613	981	387
Springfield, MA	US	1985-2006	10.34 (10.15)	D	39207	19005	6220	3014	1384
Springfield, MO	US	1985-2006	13.46 (9.88)	C	18766	9545	3609	1173	408
St. Charles, MO	US	1985-2006	14.03 (10.53)	C	11648	7113	1712	484	187
St. Louis, MO	US	1985-2006	14.53 (10.51)	C	141440	84397	21579	5279	1985
St. Petersburg, FL	US	1985-2006	23.73 (5.16)	C	110639	62599	18250	2174	1211
Stamford, CT	US	1985-2006	10.95 (9.37)	C	63213	32050	9591	3226	980
State College, PA	US	1985-2006	10.46 (9.86)	D	7326	3652	1149	537	310
Steubenville, OH	US	1985-2006	11.76 (9.96)	C	9295	5440	1181	534	149

Stockton, CA	US	1985-2006	16.36 (6.8)	C	36531	20796	7060	1344	268
Tacoma, WA	US	1985-2006	11.7 (5.74)	C	40973	18967	7930	1239	386
Tallahassee, FL	US	1985-2006	19.58 (6.93)	C	9488	4001	1988	253	149
Tampa, FL	US	1985-2006	22.57 (5.24)	C	66267	35915	11049	1135	720
Terre Haute, IN	US	1985-2006	12.06 (10.23)	C	11167	5933	1860	650	186
Toledo, OH	US	1985-2006	10.31 (10.33)	D	42975	23681	6495	1723	397
Topeka, KS	US	1985-2006	12.88 (10.73)	C	13399	6303	2485	1018	251
Trenton, NJ	US	1985-2006	12.42 (9.62)	C	24406	13800	3207	709	455
Tucson, AZ	US	1985-2006	20.85 (7.99)	B	52702	26005	8677	3560	848
Tulsa, OK	US	1985-2006	16.08 (9.92)	C	42638	23647	6949	2314	495
Upper Marlboro, MD	US	1985-2006	13.46 (9.26)	C	30995	13332	4604	1029	799
Vancouver, WA	US	1985-2006	12.17 (6.31)	C	14813	7138	2907	509	211
Ventura, CA	US	1985-2006	15.54 (4.3)	C	36364	19673	6799	1069	449
Visalia, CA	US	1985-2006	17.38 (7.58)	B	22521	12332	3990	1187	349
Washington, DC	US	1985-2006	14.38 (9.32)	C	52333	20473	7609	2028	698
Washington, PA	US	1985-2006	10.88 (9.7)	C	21443	12639	3118	1232	343
Wichita, KS	US	1985-2006	13.87 (10.58)	C	28035	12889	4805	2799	550
Wilmington, DE	US	1985-2006	12.8 (9.42)	C	30607	14115	4398	1682	591
Winston-Salem, NC	US	1985-2006	15.58 (8.6)	C	21141	9811	4497	1138	440
Worcester, MA	US	1985-2006	8.65 (9.82)	D	54939	28920	8382	3986	1812
York, PA	US	1985-2006	11.93 (9.67)	C	27507	14316	4349	1467	503
Youngstown, OH	US	1985-2006	9.74 (9.93)	C	39841	21978	5949	2130	690
Guatemala	Guatemala	2009-2018	19.39 (1.61)	C	15137	6108	3310	1636	472
San José (CR)	Costa Rica	2000-2017	22.68 (1.13)	A	9288	3278	1910	123	NA
Panama	Panama	2013-2016	28.12 (1.14)	A	9747	1593	1101	NA	NA
Montevideo	Uruguay	2001-2018	18.63 (5.64)	C	78692	19410	27890	5915	1667
Guayaquil	Ecuador	2013-2019	26.13 (1.46)	A	31793	12723	7327	452	319
Quito	Ecuador	2013-2019	15.54 (1.08)	C	16409	5071	4200	550	300
Asuncion	Paraguay	2004-2019	23.31 (5.34)	C	15371	4231	4508	1212	435
Belo Horizonte	Brazil	1997-2018	22.5 (2.62)	C	108688	25153	35267	8537	2930
Brasilia	Brazil	1997-2018	21.89 (2.13)	A	60372	15567	19116	5197	NA

Cuiaba	Brazil	1997-2018	26.41 (2.74)	A	33488	10618	11095	NA	NA
Curitiba	Brazil	1997-2018	18.18 (3.95)	C	74125	26997	21648	4160	NA
Florianopolis	Brazil	1997-2018	30.3 (2.37)	C	16899	6785	4533	NA	NA
Fortaleza	Brazil	1997-2018	27.41 (1.06)	A	85605	21333	33253	5457	NA
Maceio	Brazil	1997-2018	25.77 (1.56)	A	47755	10583	18971	4444	NA
Porto Alegre	Brazil	1997-2018	20.31 (4.93)	C	93599	32255	32265	5276	NA
Salvador	Brazil	1997-2018	25.83 (1.63)	A	92863	21544	32175	6134	NA
Sao Luis	Brazil	1997-2018	27.58 (1.44)	A	36554	9128	15134	NA	NA
Sao Paulo	Brazil	1997-2018	21.25 (3.57)	C	509204	195115	128542	26822	8181
Vitoria	Brazil	1997-2018	25.1 (2.45)	A	17146	4324	6920	NA	NA
Alfred Nzo	South Africa	1997-2013	16.59 (4.16)	C	8056	435	2840	2240	NA
Amajuba	South Africa	1997-2013	18.28 (4.76)	C	14519	1209	5325	3020	NA
Amathole	South Africa	1997-2013	17.9 (4.62)	C	32216	1702	10836	7361	118
Bojanala	South Africa	1997-2013	18.79 (4.65)	B	37425	3053	10134	11059	192
Buffalo City	South Africa	1997-2013	12.36 (3.14)	C	31330	3845	9738	4130	194
Cacadu	South Africa	1997-2013	18.5 (5.01)	B	14618	2696	4825	2615	101
Cape Winelands	South Africa	1997-2013	15.76 (5.58)	C	19915	5533	6630	2170	256
Capricorn	South Africa	1997-2013	19.32 (4.27)	B	31464	3900	8576	6749	NA
Central Karoo	South Africa	1997-2013	18.74 (5.29)	B	3045	658	899	597	NA
Chris Hani	South Africa	1997-2013	15.61 (5.51)	C	21509	1664	6751	4377	NA
City of Cape Town	South Africa	1997-2013	17.77 (4.31)	C	86707	27721	24000	7466	1125
City of Johannesburg	South Africa	1997-2013	16.44 (4.26)	C	80802	14975	19747	10138	1168
City of Tshwane	South Africa	1997-2013	16.11 (4.99)	C	74084	13467	17700	15943	606
Dr Kenneth Kaunda	South Africa	1997-2013	18.14 (5.28)	B	23051	3966	6622	3982	178
Dr Ruth Segomotsi Mompati	South Africa	1997-2013	19.97 (5.76)	B	13603	1076	4014	3458	NA
Eden	South Africa	1997-2013	17.73 (5.08)	B	19329	5711	5770	2597	240
Ehlanzeni	South Africa	1997-2013	20.39 (3.92)	C	35122	3398	14870	5496	175
Ekurhuleni	South Africa	1997-2013	16.26 (4.47)	C	61648	10344	17719	12542	544
eThekwini	South Africa	1997-2013	21.5 (3.36)	C	89200	19913	25005	12697	780
Fezile Dabi	South Africa	1997-2013	16.64 (5.48)	C	20032	2562	5465	5631	219

Frances Baard	South Africa	1997-2013	18.83 (6.07)	B	13474	2119	4279	2371	133
Gert Sibande	South Africa	1997-2013	16.36 (4.05)	C	25713	2635	7888	5898	131
Greater Sekhukhune	South Africa	1997-2013	20.38 (4.88)	B	28900	2880	9483	6638	NA
iLembe	South Africa	1997-2013	21.55 (3.41)	C	12995	1595	5702	2170	NA
Joe Gqabi	South Africa	1997-2013	16 (5.58)	B	10233	846	2865	3210	NA
John Taolo Gaetsewe	South Africa	1997-2013	18.65 (6.09)	B	4628	483	1308	958	NA
Lejweleputswa	South Africa	1997-2013	16.98 (5.67)	B	23514	3029	6615	6859	139
Mangaung	South Africa	1997-2013	16.58 (5.84)	B	22899	2997	7382	4310	297
Mopani	South Africa	1997-2013	21.75 (4.24)	B	18322	1150	7381	4089	NA
Namakwa	South Africa	1997-2013	18.88 (5.72)	B	3672	1090	942	506	NA
Nelson Mandela Bay	South Africa	1997-2013	17.77 (3.64)	C	37467	7675	11559	4262	287
Ngaka Modiri Molema	South Africa	1997-2013	19.76 (4.93)	B	27019	1515	7682	7897	NA
Nkangala	South Africa	1997-2013	16.05 (4.87)	C	33953	3849	9139	7876	162
O.R.Tambo	South Africa	1997-2013	17.2 (4.65)	C	16577	728	6085	3333	119
Overberg	South Africa	1997-2013	16.91 (4.53)	B	6648	2014	1946	943	NA
Pixley ka Seme	South Africa	1997-2013	18.06 (6.04)	B	8356	1405	2714	1661	NA
Sedibeng	South Africa	1997-2013	17.15 (5.19)	C	33019	5130	9134	7562	502
Sisonke	South Africa	1997-2013	14.18 (4.42)	C	10162	623	4291	1808	NA
Siyanda	South Africa	1997-2013	20.7 (6.53)	B	8126	1709	2266	1290	NA
Thabo Mofutsanyane	South Africa	1997-2013	14.93 (4.95)	C	32358	3021	9452	7904	237
Ugu	South Africa	1997-2013	21.21 (2.87)	C	25297	3382	11082	4276	128
uMgungundlovu	South Africa	1997-2013	13.92 (4.43)	C	33820	5460	10959	4760	221
uMkhanyakude	South Africa	1997-2013	22.53 (3.49)	A	8631	589	3786	1698	NA
uMzinyathi	South Africa	1997-2013	16.04 (4.56)	C	12584	1004	5368	2713	NA
uThukela	South Africa	1997-2013	15.51 (4.52)	C	23753	3983	8532	4506	130
uThungulu	South Africa	1997-2013	22.77 (3.92)	C	20767	1251	7664	5269	NA
Vhembe	South Africa	1997-2013	22.46 (5.15)	B	13207	840	5573	2816	NA
Waterberg	South Africa	1997-2013	21.9 (4.89)	B	10795	1915	2880	2484	NA
West Coast	South Africa	1997-2013	19.05 (5.31)	B	13653	4439	4177	1602	101
West Rand	South Africa	1997-2013	17.13 (4.52)	C	20282	3851	5577	4037	174
Xhariep	South Africa	1997-2013	17.7 (6.24)	B	8718	1216	2506	2399	NA

Zululand	South Africa	1997-2013	18.39 (4.34)	C	12471	1068	4556	3563	NA
Helsinki	Finland	1987-2018	5.93 (9.01)	D	90992	49462	23300	1201	1541
Harjumaa	Estonia	1997-2018	6.45 (8.5)	D	66103	26999	15258	582	296
Hiiumaa	Estonia	1997-2018	6.75 (8.83)	D	1568	692	354	52	NA
Ida-Virumaa	Estonia	1997-2018	5.6 (9.17)	D	30448	16188	4437	479	NA
Jõgevamaa	Estonia	1997-2018	5.6 (9.17)	D	6027	2665	1447	44	NA
Lääne-Virumaa	Estonia	1997-2018	5.6 (9.17)	D	11147	4558	2394	139	NA
Pärnumaa	Estonia	1997-2018	6.75 (8.83)	D	13103	6087	2692	143	NA
Raplamaa	Estonia	1997-2018	6.45 (8.5)	D	5032	1866	1143	135	NA
Saaremaa	Estonia	1997-2018	6.75 (8.83)	C	5622	2680	973	132	NA
Tartumaa	Estonia	1997-2018	6.38 (9.23)	D	20614	10675	2975	139	113
Accrington/Rossendale	UK	1990-2016	9.01 (5)	C	5743	3262	1366	213	NA
Barnsley/Dearne Valley	UK	1990-2016	10.03 (5.22)	C	9798	5716	2210	301	105
Basildon	UK	1990-2016	10.84 (5.44)	C	7233	3888	1613	303	123
Basingstoke	UK	1990-2016	10.32 (5.35)	C	4958	2694	1218	153	NA
Bedford	UK	1990-2016	10.61 (5.48)	C	7405	3699	2057	316	185
Birkenhead	UK	1990-2016	10.65 (4.91)	C	25434	13346	6775	852	519
Blackburn	UK	1990-2016	9.56 (5)	C	10405	5914	2494	368	130
Blackpool	UK	1990-2016	10.19 (4.82)	C	23448	11905	6101	948	381
Bournemouth/Poole	UK	1990-2016	11.04 (5.02)	C	41459	20216	12027	1536	765
Brighton and Hove	UK	1990-2016	10.74 (5.05)	C	43298	19795	11586	1216	692
Bristol	UK	1990-2016	10.94 (5.16)	C	41784	21826	10528	1104	577
Burnley	UK	1990-2016	9.16 (5.01)	C	11472	6360	2715	437	130
Burton upon Trent	UK	1990-2016	10.06 (5.32)	C	6004	3318	1394	202	NA
Cambridge	UK	1990-2016	10.84 (5.58)	C	7645	3584	2108	253	186
Cardiff	UK	1990-2016	10.92 (5.1)	C	27309	14013	7198	776	445
Chelmsford	UK	1990-2016	10.62 (5.53)	C	5449	2788	1337	252	NA
Cheltenham	UK	1990-2016	10.82 (5.43)	C	9450	4570	2689	317	141
Chesterfield	UK	1990-2016	9.9 (5.24)	C	7028	3700	1789	208	138
Colchester	UK	1990-2016	10.71 (5.52)	C	6068	2911	1648	370	142
Coventry	UK	1990-2016	10.27 (5.41)	C	24435	12058	6241	1002	485

Crawley	UK	1990-2016	10.5 (5.42)	C	8919	4107	2285	303	181
Derby	UK	1990-2016	10.35 (5.3)	C	18304	10018	4312	543	267
Doncaster	UK	1990-2016	10.41 (5.32)	C	9032	4811	2142	218	171
Eastbourne	UK	1990-2016	11.12 (5.01)	C	9831	4231	2198	224	122
Exeter	UK	1990-2016	10.91 (4.82)	C	7542	3777	2131	261	135
Farnborough/Aldershot	UK	1990-2016	10.56 (5.45)	C	11198	5335	2977	494	186
Gloucester	UK	1990-2016	10.99 (5.35)	C	9754	5233	2636	214	NA
Grimsby	UK	1990-2016	10.33 (5.14)	C	9129	5289	2170	288	154
Hastings	UK	1990-2016	10.87 (5.05)	C	15018	6531	3985	503	161
High Wycombe	UK	1990-2016	10.5 (5.44)	C	4737	2354	1257	188	NA
Ipswich	UK	1990-2016	10.64 (5.5)	C	11825	5837	3252	377	236
Kingston upon Hull	UK	1990-2016	10.55 (5.22)	C	24252	13262	6359	760	454
Leicester	UK	1990-2016	10.04 (5.37)	C	32384	17355	7872	1169	562
Lincoln	UK	1990-2016	9.95 (5.38)	C	6729	3546	1752	314	112
Liverpool	UK	1990-2016	10.59 (5.04)	C	73227	40301	17621	2834	1291
London	UK	1990-2016	11.41 (5.49)	C	588591	293931	141928	23777	8507
Luton	UK	1990-2016	10.2 (5.53)	C	15855	8296	3750	625	336
Maidstone	UK	1990-2016	10.67 (5.43)	C	5959	2951	1528	237	NA
Manchester	UK	1990-2016	10.13 (5.12)	C	193740	104864	50078	5583	2745
Mansfield	UK	1990-2016	9.79 (5.28)	C	9670	4984	2436	462	223
Medway Towns	UK	1990-2016	11.06 (5.51)	C	15056	7849	3354	410	291
Milton Keynes	UK	1990-2016	10.36 (5.43)	C	7585	3871	1847	302	134
Newport	UK	1990-2016	10.46 (5.07)	C	15629	8206	3582	596	237
Northampton	UK	1990-2016	10.38 (5.44)	C	13345	6613	3252	520	221
Norwich	UK	1990-2016	10.31 (5.41)	C	14154	7056	3749	508	298
Nottingham	UK	1990-2016	10.39 (5.32)	C	49017	25041	13054	1831	914
Oxford	UK	1990-2016	10.83 (5.47)	C	6721	3245	1915	227	124
Paignton/Torquay	UK	1990-2016	11.25 (4.52)	C	11969	5604	3449	509	194
Peterborough	UK	1990-2016	10.54 (5.54)	C	9925	5213	2445	406	157
Plymouth	UK	1990-2016	10.99 (4.54)	C	19478	9929	4829	650	315
Preston	UK	1990-2016	10.13 (5.02)	C	16874	8975	4336	629	183

Reading	UK	1990-2016	10.92 (5.4)	C	14536	7096	3942	448	287
Sheffield	UK	1990-2016	10 (5.25)	C	47778	26160	12299	1241	585
Slough	UK	1990-2016	11.07 (5.49)	C	8143	4610	1850	282	104
South Hampshire	UK	1990-2016	11.41 (5.15)	C	54375	27107	14201	2058	946
Southend-on-Sea	UK	1990-2016	11.21 (5.54)	C	24133	11661	6194	999	360
Stoke-on-Trent	UK	1990-2016	9.49 (5.16)	C	26187	14036	6173	854	517
Sunderland	UK	1990-2016	9.76 (4.73)	C	22329	12587	5530	632	296
Swansea	UK	1990-2016	11.16 (4.72)	C	16556	8708	4303	582	330
Swindon	UK	1990-2016	10.4 (5.33)	C	10705	5846	2374	332	189
Teesside	UK	1990-2016	9.89 (5.01)	C	28125	15571	7114	821	375
Telford	UK	1990-2016	9.86 (5.22)	C	5995	3345	1437	181	NA
Thanet	UK	1990-2016	11.26 (5.23)	C	10444	4981	2828	284	140
Tyneside	UK	1990-2016	9.73 (4.8)	C	63000	33746	15972	1680	868
Warrington	UK	1990-2016	10.49 (5.17)	C	10693	5793	2548	325	182
West Midlands	UK	1990-2016	10.14 (5.32)	C	202529	105863	52123	8132	4376
West Yorkshire	UK	1990-2016	9.8 (5.16)	C	115432	62690	29635	3921	1724
Wigan	UK	1990-2016	10.09 (5.05)	C	10114	5594	2440	401	181
Worcester	UK	1990-2016	10.73 (5.41)	C	6074	3001	1568	219	109
York	UK	1990-2016	10.16 (5.33)	C	9874	5298	2482	248	133
Basel	Switzerland	1995-2016	10.92 (7.36)	C	17618	7643	3064	1014	387
Bern	Switzerland	1995-2016	9.43 (7.5)	C	79437	32229	14518	6530	1690
Geneve	Switzerland	1995-2016	11.09 (7.36)	C	19475	5825	4386	3284	790
Lucerne	Switzerland	1995-2016	10.05 (7.44)	C	24919	10967	4499	1996	387
St. Gallen	Switzerland	1995-2016	8.67 (7.56)	C	34355	14789	6145	2551	534
Ticino	Switzerland	1995-2016	12.99 (7.07)	D	22002	9238	4134	1491	414
Vaud	Switzerland	1995-2016	11.38 (7.16)	C	38205	12433	6830	6229	1622
Zürich	Switzerland	1995-2016	9.76 (7.48)	C	85646	37032	15669	7028	1661
Chisinau	Moldova	2001-2010	10.79 (9.8)	C	33087	22062	9830	NA	NA
Beja	Portugal	1990-2018	16.59 (6.07)	C	28600	7351	12193	3578	526
Castelo Branco	Portugal	1990-2018	15.77 (6.83)	C	31752	5342	16019	4600	680
Coimbra	Portugal	1990-2018	15.19 (4.85)	C	53349	7506	26911	10944	1309

Faro	Portugal	1990-2018	18.14 (4.84)	C	44460	10577	20869	4284	858
Lisboa	Portugal	1990-2018	17.04 (4.91)	C	237952	74837	95636	15906	3380
Porto	Portugal	1990-2018	14.89 (4.33)	C	135746	26443	69924	18474	2692
A Coruna	Spain	2000-2018	15.15 (3.89)	C	14650	2085	3911	2446	591
Barcelona	Spain	2000-2018	16.87 (6.1)	C	89450	24933	21751	12798	4788
Madrid	Spain	2000-2018	15.54 (7.69)	C	119519	36068	26297	22030	4513
Malaga	Spain	2000-2018	19.11 (5.55)	C	38657	10175	10855	5563	1349
Sevilla	Spain	2000-2018	19.69 (6.77)	C	59510	16942	18226	9869	1314
Zaragoza	Spain	2000-2018	16.07 (7.55)	B	34816	9607	9255	5547	1791
Civitavecchia	Italy	2006-2015	18.03 (5.96)	C	2588	886	494	299	NA
Frosinone	Italy	2006-2015	15.64 (7.55)	C	4122	1050	1096	317	153
Latina	Italy	2006-2015	17.31 (6.92)	C	5867	1516	2021	357	117
Rieti	Italy	2006-2015	16.96 (8.22)	C	3245	974	957	110	NA
Rome	Italy	2006-2015	16.02 (6.97)	C	101162	34977	21428	3923	3868
Viterbo	Italy	2006-2015	15.1 (7.41)	C	4821	1357	1483	275	257
Ammochostos	Cyprus	2004-2017	20.14 (6.34)	B	1187	410	234	80	NA
Larnaca	Cyprus	2004-2017	20.32 (6.04)	B	4355	1410	915	578	152
Limassol	Cyprus	2004-2017	21.3 (5.82)	C	8058	2675	1568	1331	351
Nicosia	Cyprus	2004-2017	20.34 (7.38)	C	9999	3191	2200	1456	430
Pafos	Cyprus	2004-2017	19.91 (5.11)	C	3030	969	500	376	174
Mashhad	Iran	2004-2013	9.88 (8)	B	41389	15121	12066	9328	4885
Tehran	Iran	2001-2017	17.47 (9.9)	B	376367	105659	59246	45766	8831
Kuwait	Kuwait	2000-2016	27.1 (9.82)	B	35285	17251	6615	4620	234
Ayutthaya	Thailand	1999-2008	28.37 (2.13)	A	5899	2035	2089	673	246
Bangkok	Thailand	1999-2008	29.25 (1.73)	A	50321	17852	19449	783	1734
Buri Ram	Thailand	1999-2008	26.87 (2.71)	A	4941	1057	1915	825	206
Chachoengsao	Thailand	1999-2008	26.15 (2.99)	A	4422	1089	1690	766	NA
Chaiyaphum	Thailand	1999-2008	27.75 (2.53)	A	3428	740	1036	563	115
Chanthaburi	Thailand	1999-2008	27.34 (1.45)	A	4751	1222	2178	461	140
Chiang Mai	Thailand	1999-2008	26.35 (2.61)	A	11746	3081	4352	456	360
Chiang Rai	Thailand	1999-2008	25.08 (3.07)	A	7424	1668	2451	349	177

Chon Buri	Thailand	1999-2008	28.37 (1.44)	A	9090	2825	3856	66	367
Chumphon	Thailand	1999-2008	28.61 (1.9)	A	2536	766	981	301	101
Kalasin	Thailand	1999-2008	27.7 (2.83)	A	3382	780	896	712	NA
Kamphaeng Phet	Thailand	1999-2008	26.39 (3.1)	A	2937	1017	969	350	NA
Khon Kaen	Thailand	1999-2008	27.2 (2.79)	A	9406	2213	3134	1268	430
Krabi	Thailand	1999-2008	27.78 (1.24)	A	1467	486	432	182	NA
Lampang	Thailand	1999-2008	27.96 (2.26)	A	6664	1511	2288	278	184
Lamphun	Thailand	1999-2008	26.19 (2.81)	A	2696	582	800	225	NA
Lop Buri	Thailand	1999-2008	27.33 (1.19)	A	5669	1681	2237	450	170
Maha Sarakham	Thailand	1999-2008	28.84 (1.83)	A	3403	655	875	999	NA
Mukdahan	Thailand	1999-2008	27.76 (1.32)	A	1010	181	221	309	NA
Nakhon Pathom	Thailand	1999-2008	27.93 (2.37)	A	4928	1465	2200	88	NA
Nakhon Phanom	Thailand	1999-2008	27.8 (1.22)	A	2126	426	535	726	NA
Nakhon Ratchasima	Thailand	1999-2008	27.09 (2.44)	A	13929	3447	6731	130	582
Nakhon Sawan	Thailand	1999-2008	28.48 (2.23)	A	9325	2400	4667	658	261
Nakhon Si Thammarat	Thailand	1999-2008	27.33 (1.18)	A	9239	3629	3137	881	204
Nan	Thailand	1999-2008	26.65 (2.93)	A	3325	896	1164	181	109
Narathiwat	Thailand	1999-2008	27.72 (1.91)	A	4780	830	624	882	NA
Nong Khai	Thailand	1999-2008	27.91 (2.37)	A	2701	500	660	812	NA
Pathum Thani	Thailand	1999-2008	26.96 (3.07)	A	5068	1497	1922	380	305
Pattani	Thailand	1999-2008	27.81 (1.27)	A	3166	622	484	484	NA
Phayao	Thailand	1999-2008	27.16 (2.82)	A	3729	584	816	611	NA
Phetchabun	Thailand	1999-2008	27.13 (1.14)	A	5060	1448	1504	896	233
Phetchaburi	Thailand	1999-2008	27.96 (1.07)	A	2890	890	1079	307	NA
Phichit	Thailand	1999-2008	27.79 (1.62)	A	3208	843	1279	417	197
Phitsanulok	Thailand	1999-2008	28.33 (1.92)	A	7465	2020	3759	30	258
Phrae	Thailand	1999-2008	28.14 (2.13)	A	3766	811	1097	433	NA
Prachin Buri	Thailand	1999-2008	28.41 (1.09)	A	2976	602	1555	203	NA
Prachuap Khiri Khan	Thailand	1999-2008	27.89 (1.37)	A	2693	889	857	322	NA
Ratchaburi	Thailand	1999-2008	26.79 (2.8)	A	6107	1938	2391	418	175
Rayong	Thailand	1999-2008	25.82 (2.45)	A	3655	697	1710	624	NA

Roi Et	Thailand	1999-2008	27.15 (2.9)	A	4615	869	1497	758	139
Sa Kaeo	Thailand	1999-2008	27.67 (1.72)	A	2195	826	602	303	NA
Sakon Nakhon	Thailand	1999-2008	26.46 (3.2)	A	3858	759	1092	1030	NA
Samutprakan	Thailand	1999-2008	28.32 (1.54)	A	6833	1889	2469	856	118
Si Sa Ket	Thailand	1999-2008	27.31 (2.68)	A	4509	1176	1240	881	198
Songkhla	Thailand	1999-2008	27.93 (1.17)	A	7273	2521	2674	NA	244
Sukhothai	Thailand	1999-2008	28.31 (2.07)	A	3420	947	980	517	NA
Suphanburi	Thailand	1999-2008	28.08 (2.09)	A	5808	1560	2079	883	130
Surat Thani	Thailand	1999-2008	27.34 (2.86)	A	3890	1105	1385	54	115
Surin	Thailand	1999-2008	27.5 (2.67)	A	4983	925	1965	898	176
Tak	Thailand	1999-2008	28.02 (1.2)	A	2146	718	665	295	NA
Trang	Thailand	1999-2008	28.67 (1.67)	A	3266	1255	884	474	NA
Ubon Ratchathani	Thailand	1999-2008	27.53 (2.55)	A	7675	1911	2962	767	218
Udon Thani	Thailand	1999-2008	27.13 (3.11)	A	5395	1225	2039	315	143
Uttaradit	Thailand	1999-2008	27.3 (3.03)	A	4825	1422	1705	276	NA
Yala	Thailand	1999-2008	27.6 (0.97)	A	3086	718	961	354	NA
Cebu	Philippines	2006-2010	28.15 (1.16)	A	13365	4174	5283	469	114
Davao	Philippines	2006-2010	28.1 (1)	A	15924	2867	6714	805	118
Manila	Philippines	2006-2010	28.78 (1.52)	A	26615	13041	9042	357	128
Quezon	Philippines	2006-2010	27.97 (1.57)	A	31497	14324	9139	987	567
Ho Chi Minh City	Vietnam	2010-2013	28.45 (1.36)	A	22196	3607	9426	3872	NA
Andong	South Korea	1997-2018	12.35 (10)	D	7852	1346	4331	NA	NA
Boryeong	South Korea	1997-2018	12.91 (9.51)	C	4142	904	2115	NA	NA
Busan	South Korea	1997-2018	15.04 (8.15)	C	114135	29705	50124	NA	NA
Cheonan	South Korea	1997-2018	12.11 (10.24)	D	10110	2055	5060	NA	NA
Chuncheon	South Korea	1997-2018	11.53 (10.85)	D	7968	1798	4186	NA	NA
Chungju	South Korea	1997-2018	11.87 (10.58)	D	7303	1427	3722	NA	NA
Daegu	South Korea	1997-2018	14.56 (9.5)	C	62767	15390	30681	NA	NA
Daejeon	South Korea	1997-2018	13.2 (10.03)	D	29193	6053	15346	NA	NA
Donghae	South Korea	1997-2018	12.96 (8.52)	D	3218	688	1739	NA	NA
Gangneung	South Korea	1997-2018	13.53 (9.17)	D	7453	1748	3830	NA	NA

Geojae	South Korea	1997-2018	14.49 (8.41)	C	5297	1157	2498	NA	NA
Gumi	South Korea	1997-2018	13.3 (9.7)	D	6500	1250	3493	NA	NA
Gwangju	South Korea	1997-2018	14.28 (9.42)	C	28395	5975	14403	NA	NA
Icheon	South Korea	1997-2018	11.7 (10.45)	D	4762	933	2552	NA	NA
Incheon	South Korea	1997-2018	12.65 (9.95)	C	61894	13672	32672	NA	NA
Jecheon	South Korea	1997-2018	10.42 (10.64)	D	5058	981	2667	NA	NA
Jeju	South Korea	1997-2018	16.26 (7.72)	C	7466	1498	3528	NA	NA
Jeongeup	South Korea	1997-2018	13.47 (9.76)	C	6110	957	3592	NA	NA
Jinju	South Korea	1997-2018	13.52 (9.36)	C	10052	2524	4407	NA	NA
Milyang	South Korea	1997-2018	13.79 (9.41)	C	6030	1275	2871	NA	NA
Mokpo	South Korea	1997-2018	14.01 (8.97)	C	5762	1148	2900	NA	NA
Mungyeong	South Korea	1997-2018	11.94 (9.66)	D	4341	1085	2002	NA	NA
Namwon	South Korea	1997-2018	12.6 (9.98)	D	4262	696	2317	NA	NA
Pohang	South Korea	1997-2018	14.65 (8.86)	C	13807	2928	6965	NA	NA
Seogyupo	South Korea	1997-2018	17.08 (7.27)	C	3329	679	1564	NA	NA
Seosan	South Korea	1997-2018	12.15 (9.88)	C	4052	680	2241	NA	NA
Seoul	South Korea	1997-2018	12.95 (10.49)	D	192817	44110	99713	NA	NA
Sokcho	South Korea	1997-2018	12.59 (8.97)	D	2690	463	1427	NA	NA
Suwon	South Korea	1997-2018	12.65 (10.39)	C	18219	3622	9597	NA	NA
Taebaek	South Korea	1997-2018	9.14 (9.82)	D	1702	379	884	NA	NA
Tongyeong	South Korea	1997-2018	14.72 (8.16)	C	4754	1109	2303	NA	NA
Ulsan	South Korea	1997-2018	14.53 (8.66)	C	22222	4698	10691	NA	NA
Wonju	South Korea	1997-2018	12.14 (10.62)	D	8550	1548	4266	NA	NA
Yeongju	South Korea	1997-2018	11.75 (9.89)	D	5171	1003	2660	NA	NA
Yeosu	South Korea	1997-2018	14.66 (8.38)	C	8662	2000	4066	NA	NA
Yoengcheon	South Korea	1997-2018	12.89 (9.6)	C	5593	1153	2724	NA	NA
Aichi	Japan	1979-2015	15.89 (8.46)	C	539638	105549	219844	127561	18687
Akita	Japan	1979-2015	11.79 (9)	C	150041	21599	72902	31211	5795
Aomori	Japan	1979-2015	10.43 (8.93)	C	162993	28853	72816	37221	5481
Chiba	Japan	1979-2015	15.84 (7.7)	C	454458	84856	182422	106402	11709
Ehime	Japan	1979-2015	16.48 (7.98)	C	174295	28383	69313	49191	4133

Fukui	Japan	1979-2015	14.61 (8.79)	C	86436	17411	35997	19970	2662
Fukuoka	Japan	1979-2015	17 (7.83)	C	415867	82918	173475	83910	13680
Fukushima	Japan	1979-2015	13.1 (8.76)	D	244827	48044	112050	48775	7492
Gifu	Japan	1979-2015	15.9 (8.57)	C	206279	35198	87439	51304	6368
Gunma	Japan	1979-2015	14.67 (8.47)	C	204612	34464	90698	41109	5364
Hiroshima	Japan	1979-2015	16.12 (8.32)	C	271069	54141	109855	66958	6417
Hokkaido	Japan	1979-2015	9 (9.51)	D	519006	104700	200549	124638	15284
Hyogo	Japan	1979-2015	16.44 (8.2)	C	480145	98198	186526	118359	13282
Ibaraki	Japan	1979-2015	13.78 (8.12)	C	287160	57595	130723	58070	8232
Ishikawa	Japan	1979-2015	14.72 (8.55)	C	115947	21708	49074	24779	3681
Iwate	Japan	1979-2015	10.34 (9.28)	D	169259	28162	78399	32343	4720
Kagawa	Japan	1979-2015	16.33 (8.27)	C	110254	21435	43094	25388	3052
Kagoshima	Japan	1979-2015	18.46 (7.47)	C	220610	37041	98634	48875	6461
Kanagawa	Japan	1979-2015	15.89 (7.6)	C	564892	126753	238366	111314	15295
Kochi	Japan	1979-2015	16.99 (7.74)	C	108767	20417	47601	25451	3007
Kumamoto	Japan	1979-2015	16.94 (8.24)	C	196754	34139	79433	41862	6547
Kyoto	Japan	1979-2015	15.93 (8.62)	C	241274	55205	94006	53785	6653
Mie	Japan	1979-2015	15.96 (8.15)	C	193256	39054	82339	39601	6131
Miyagi	Japan	1979-2015	12.49 (8.27)	C	218311	39246	102453	38164	7434
Miyazaki	Japan	1979-2015	17.62 (7.45)	C	127877	24125	54185	27031	3615
Nagano	Japan	1979-2015	12.01 (9.45)	D	263305	42606	128781	51970	7673
Nagasaki	Japan	1979-2015	17.19 (7.6)	C	165339	32727	66764	33175	5038
Nara	Japan	1979-2015	14.94 (8.44)	C	126640	20743	48811	30563	3003
Niigata	Japan	1979-2015	13.89 (8.65)	C	277734	44744	135072	53350	8562
Oita	Japan	1979-2015	16.43 (7.73)	C	140571	31448	59398	26956	3654
Okayama	Japan	1979-2015	16.1 (8.56)	C	205106	35338	89374	53623	5095
Okinawa	Japan	1979-2015	23.05 (4.71)	C	77441	18026	28698	14458	2975
Osaka	Japan	1979-2015	16.9 (8.29)	C	669549	175187	239997	144503	16343
Saga	Japan	1979-2015	16.62 (8.16)	C	93549	16904	39223	18101	2956
Saitama	Japan	1979-2015	15.07 (8.35)	C	481658	118633	196621	97032	14135
Shiga	Japan	1979-2015	14.77 (8.52)	C	114114	21366	46107	27449	3748

Shimane	Japan	1979-2015	14.92 (8.16)	C	96770	14334	43103	21683	2393
Shizuoka	Japan	1979-2015	16.63 (7.4)	C	350252	62510	152191	76381	10784
Tochigi	Japan	1979-2015	13.92 (8.49)	C	206077	46508	96767	34201	5281
Tokushima	Japan	1979-2015	16.59 (7.93)	C	95822	18446	39473	21075	2886
Tokyo	Japan	1979-2015	16.34 (7.82)	C	968025	266560	400586	147382	27020
Tottori	Japan	1979-2015	14.95 (8.44)	C	73800	12801	33397	16527	2010
Toyama	Japan	1979-2015	14.13 (8.75)	C	117377	20575	53696	25343	3399
Wakayama	Japan	1979-2015	16.68 (8.07)	C	126706	25908	49568	33898	3257
Yamagata	Japan	1979-2015	11.8 (9.28)	D	155159	26825	73857	31561	4770
Yamaguchi	Japan	1979-2015	15.44 (8.37)	C	180675	31144	76056	36488	4839
Yamanashi	Japan	1979-2015	14.74 (8.64)	D	92201	16411	40442	20680	2802
Kaohsiung	Taiwan	2008-2016	25.44 (4.06)	A	18907	5125	5801	1941	618
Taichung	Taiwan	2008-2016	23.6 (5.19)	C	12991	2890	3864	1664	505
Taipei	Taiwan	2008-2016	23.11 (5.55)	C	75312	20532	23043	6622	3349

Climate zones are classified according to the Koppen-Geiger climate classification (DOI: 10.1127/0941-2948/2006/0130); where the five main climate groups are: A (tropical), B (dry), C (temperate), D (continental), and E (polar).

Table S4. Relative risk of death for extreme cold (1st vs. MMT) and extreme heat (99th vs. MMT) across all countries

		**All CVD**		**Ischemic Heart Disease**		**Stroke**		**Heart Failure**		**Arrythmia**	
	**Country**	**Cold**	**Heat**	**Cold**	**Heat**	**Cold**	**Heat**	**Cold**	**Heat**	**Cold**	**Heat**
**North America**											
**1**	Canada	1.32 [1.24,1.42]	1.12 [1.08,1.16]	1.39 [1.28,1.51]	1.1 [1.06,1.14]	1.29 [1.2,1.38]	1.1 [1.05,1.16]	1.32 [1.18,1.47]	1.16 [1.06,1.26]	1.08 [0.9,1.3]	1.08 [0.99,1.18]
**2**	US	1.36 [1.29,1.44]	1.2 [1.14,1.27]	1.4 [1.3,1.5]	1.18 [1.13,1.24]	1.3 [1.23,1.38]	1.12 [1.04,1.2]	1.37 [1.25,1.5]	1.19 [1.04,1.36]	1.28 [1.15,1.42]	0.99 [0.9,1.09]
**Caribbean and Central America**											
**3**	Guatemala	1.29 [1.17,1.43]	1.02 [0.99,1.04]	1.34 [1.18,1.51]	1.01 [0.98,1.03]	1.33 [1.19,1.49]	1.04 [1,1.07]	1.26 [1.12,1.43]	1.01 [0.94,1.09]	1.2 [0.83,1.73]	1.07 [0.96,1.2]
**4**	Costa Rica	1.32 [1.24,1.41]	1.05 [1.02,1.08]	1.33 [1.23,1.45]	1.03 [1,1.07]	1.29 [1.21,1.39]	1.07 [1.03,1.12]	1.29 [1.18,1.42]	1.03 [0.95,1.11]	NA	NA
**5**	Panama	1.46 [1.36,1.56]	1.19 [1.08,1.3]	1.44 [1.32,1.58]	1.15 [1.04,1.27]	1.4 [1.29,1.52]	1.15 [1.01,1.3]	NA	NA	NA	NA
**South America**											
**6**	Uruguay	1.38 [1.29,1.46]	1.12 [1.07,1.16]	1.38 [1.27,1.49]	1.08 [1.04,1.12]	1.38 [1.29,1.48]	1.12 [1.05,1.18]	1.42 [1.29,1.55]	1.12 [1.04,1.22]	1.26 [1.05,1.51]	1.01 [0.92,1.12]
**7**	Ecuador	1.2 [1.14,1.26]	1.17 [1.07,1.27]	1.19 [1.11,1.28]	1.12 [1.02,1.22]	1.17 [1.1,1.24]	1.2 [1.07,1.35]	1.17 [1.09,1.25]	1.2 [0.98,1.46]	1.14 [0.98,1.33]	0.89 [0.66,1.19]
**8**	Paraguay	1.42 [1.31,1.54]	1.1 [1.04,1.16]	1.4 [1.26,1.55]	1.08 [1.03,1.13]	1.44 [1.32,1.58]	1.1 [1.02,1.17]	1.49 [1.32,1.68]	1.06 [0.99,1.15]	1.36 [1.07,1.74]	1.03 [0.91,1.17]
**9**	Brazil	1.38 [1.29,1.49]	1.08 [1.03,1.12]	1.34 [1.24,1.46]	1.05 [1.01,1.1]	1.31 [1.22,1.4]	1.1 [1.03,1.17]	1.27 [1.15,1.41]	1.08 [0.98,1.18]	1.25 [1.04,1.5]	1.04 [0.92,1.19]
**South Africa**											
**10**	South Africa	1.31 [1.22,1.41]	1.11 [1.05,1.18]	1.32 [1.21,1.45]	1.07 [1.02,1.13]	1.34 [1.23,1.45]	1.15 [1.06,1.24]	1.34 [1.2,1.51]	1.15 [1.03,1.28]	1.16 [0.96,1.41]	1.03 [0.88,1.2]
**North Europe**											
**11**	Finland	1.28 [1.21,1.36]	1.12 [1.07,1.17]	1.36 [1.25,1.47]	1.1 [1.07,1.15]	1.22 [1.14,1.3]	1.09 [1.03,1.16]	1.24 [1.1,1.4]	1.16 [1.04,1.29]	1.08 [0.93,1.26]	1.07 [0.95,1.2]
**12**	Estonia	1.24 [1.16,1.32]	1.2 [1.13,1.28]	1.3 [1.19,1.42]	1.15 [1.07,1.23]	1.27 [1.17,1.38]	1.23 [1.13,1.34]	1.25 [1.08,1.44]	1.38 [1.16,1.64]	1.01 [0.82,1.25]	1.14 [0.97,1.34]
**13**	UK	1.29 [1.21,1.38]	1.1 [1.06,1.15]	1.38 [1.27,1.49]	1.09 [1.05,1.13]	1.21 [1.12,1.31]	1.1 [1.03,1.17]	1.21 [1.06,1.37]	1.13 [1.02,1.25]	1.1 [0.94,1.29]	1.03 [0.91,1.18]
**Central Europe**											
**14**	Switzerland	1.37 [1.23,1.53]	1.2 [1.07,1.35]	1.47 [1.27,1.71]	1.24 [1.12,1.38]	1.21 [1.06,1.39]	1.04 [0.87,1.25]	1.29 [1.05,1.58]	1.13 [0.86,1.49]	1.26 [0.97,1.64]	0.94 [0.71,1.25]
**15**	Moldova	1.27 [1.17,1.37]	1.11 [1.05,1.18]	1.26 [1.13,1.41]	1.05 [0.99,1.12]	1.36 [1.24,1.5]	1.16 [1.07,1.25]	NA	NA	NA	NA
**South Europe**											

**16**	Portugal	1.32 [1.26,1.38]	1.08 [1.05,1.11]	1.34 [1.26,1.42]	1.06 [1.03,1.08]	1.29 [1.23,1.36]	1.09 [1.04,1.13]	1.32 [1.22,1.42]	1.08 [1.02,1.14]	1.2 [1.06,1.35]	1.04 [0.95,1.13]
**17**	Spain	1.36 [1.3,1.43]	1.16 [1.11,1.21]	1.38 [1.3,1.46]	1.12 [1.09,1.17]	1.32 [1.26,1.39]	1.12 [1.06,1.19]	1.38 [1.29,1.49]	1.16 [1.06,1.27]	1.26 [1.14,1.4]	0.98 [0.9,1.06]
**18**	Italy	1.41 [1.33,1.48]	1.12 [1.08,1.16]	1.42 [1.33,1.51]	1.09 [1.06,1.12]	1.36 [1.29,1.44]	1.1 [1.05,1.14]	1.42 [1.32,1.53]	1.13 [1.05,1.21]	1.25 [1.12,1.4]	1.04 [0.99,1.09]
**19**	Cyprus	1.47 [1.39,1.56]	1.13 [1.08,1.18]	1.46 [1.35,1.57]	1.1 [1.06,1.14]	1.42 [1.33,1.52]	1.1 [1.04,1.16]	1.49 [1.36,1.62]	1.1 [1.01,1.19]	1.36 [1.18,1.56]	0.98 [0.91,1.07]
**Middle–East Asia**											
**20**	Iran	1.31 [1.23,1.4]	1.08 [1.04,1.14]	1.29 [1.17,1.41]	1.05 [1,1.1]	1.38 [1.27,1.49]	1.1 [1.04,1.17]	1.46 [1.27,1.68]	1.12 [1.02,1.23]	1.19 [0.98,1.44]	1.08 [0.96,1.21]
**21**	Kuwait	1.67 [1.52,1.83]	1.17 [1.08,1.27]	1.56 [1.38,1.76]	1.14 [1.04,1.24]	1.61 [1.43,1.82]	1.07 [0.96,1.18]	1.83 [1.48,2.26]	1.05 [0.84,1.32]	1.72 [1.16,2.55]	0.94 [0.73,1.21]
**East Asia**											
**22**	South Korea	1.37 [1.3,1.43]	1.15 [1.1,1.19]	1.37 [1.29,1.46]	1.11 [1.07,1.15]	1.36 [1.29,1.43]	1.12 [1.07,1.18]	NA	NA	NA	NA
**23**	Japan	1.38 [1.32,1.45]	1.17 [1.13,1.22]	1.39 [1.31,1.48]	1.13 [1.09,1.18]	1.35 [1.29,1.41]	1.13 [1.07,1.19]	1.44 [1.34,1.56]	1.2 [1.08,1.33]	1.27 [1.15,1.41]	1.01 [0.96,1.07]
**24**	Taiwan	1.41 [1.34,1.48]	1.2 [1.12,1.28]	1.39 [1.31,1.49]	1.15 [1.08,1.23]	1.37 [1.29,1.45]	1.14 [1.04,1.24]	1.44 [1.31,1.58]	1.12 [0.95,1.31]	1.41 [1.22,1.64]	0.85 [0.7,1.03]
**South East Asia**											
**25**	Thailand	1.35 [1.26,1.44]	1.14 [1.06,1.22]	1.33 [1.22,1.45]	1.11 [1.03,1.18]	1.33 [1.24,1.43]	1.14 [1.04,1.25]	1.35 [1.24,1.48]	1.07 [0.94,1.22]	1.32 [1.11,1.58]	0.99 [0.83,1.18]
**26**	Philippines	1.29 [1.21,1.37]	1.18 [1.06,1.32]	1.27 [1.17,1.38]	1.13 [1,1.26]	1.26 [1.19,1.35]	1.19 [1.03,1.38]	1.29 [1.18,1.4]	1.15 [0.89,1.5]	1.29 [1.08,1.54]	0.74 [0.5,1.09]
**27**	Vietnam	1.4 [1.3,1.52]	1.24 [1.05,1.48]	1.38 [1.25,1.53]	1.13 [0.93,1.38]	1.39 [1.28,1.52]	1.25 [0.99,1.58]	1.43 [1.28,1.59]	1.27 [0.8,2.01]	NA	NA
**Total**											
	**Total**	1.32 [1.27,1.38]	1.11 [1.07,1.14]	1.33 [1.26,1.41]	1.07 [1.04,1.1]	1.32 [1.26,1.38]	1.1 [1.06,1.15]	1.37 [1.28,1.47]	1.12 [1.05,1.19]	1.19 [1.07,1.33]	1.05 [0.98,1.12]

Table S5. Excess deaths (per 1000 deaths) attributable to a range of extreme cold temperatures (2.5th and below) and a range of extreme hot temperatures (97.5th and above)

		**All CVD**		**Ischemic Heart Disease**		**Stroke**		**Heart Failure**		**Arrythmia**	
	**Country**	**Cold**	**Heat**	**Cold**	**Heat**	**Cold**	**Heat**	**Cold**	**Heat**	**Cold**	**Heat**
**North America**											
**1**	Canada	5.75 [4.91,6.44]	1.8 [1.31,2.22]	5.71 [4.54,6.58]	1.75 [1.21,2.17]	6.83 [5.8,7.62]	1.75 [1.24,2.19]	7 [5.17,8.28]	2.49 [1.3,3.38]	2.23 [-1.72,4.35]	1.77 [0.58,2.7]
**2**	US	6.17 [5.86,6.38]	2.06 [1.85,2.22]	6.38 [5.93,6.67]	2.03 [1.76,2.21]	5.6 [5.24,5.84]	1.11 [0.88,1.27]	6.83 [6.15,7.22]	0.09 [-0.21,0.28]	3.69 [2.22,4.07]	0.53 [0,0.8]
**Caribbean and Central America**											
**3**	Guatemala	5.68 [0.44,9.58]	0.32 [-1.12,1.58]	4.5 [-2.65,10.33]	0.31 [-0.72,1.37]	6.11 [-0.58,10.91]	0.59 [-2.29,2.98]	5.49 [0.42,9.87]	1.05 [-3.36,4.51]	3.77 [-15.12,13.58]	0.83 [-1.75,2.87]
**4**	Costa Rica	3.98 [0.27,7.12]	0.27 [-2.02,2.13]	8.86 [3.66,12.34]	2.03 [-0.88,4.64]	2.2 [-1.82,5.55]	0.83 [-5.09,5.51]	7.73 [0.13,13.47]	0.72 [-2.73,3.41]	NA	NA
**5**	Panama	6.16 [-0.13,10.98]	2.93 [-3.28,7.73]	7.28 [-0.53,13.16]	2.31 [-1.89,5.89]	7.93 [1.44,12.45]	3.22 [-5.29,9.41]	NA	NA	NA	NA
**South America**											
**6**	Uruguay	11.11 [5.55,15.39]	5.39 [1.98,8.35]	11.45 [3.14,17.59]	2.63 [-1.73,5.81]	9.68 [2.68,14.86]	5.24 [0.14,9.18]	14.04 [2.62,21.87]	5.16 [-1.14,9.41]	6.73 [-18.72,19.21]	-0.6 [-6.1,3.26]
**7**	Ecuador	4.76 [1.56,7.38]	4.06 [1.67,6.41]	2.99 [-0.98,6.16]	2.58 [-0.44,5.31]	7.72 [4.31,10.63]	4.35 [1.18,7.37]	5.61 [1.85,8.55]	1.23 [-2.74,3.98]	6.05 [- 4.04,12.25]	-0.18 [-4.07,2.33]
**8**	Paraguay	10.68 [5.75,14.65]	3.61 [0.13,6.99]	10.26 [3.94,15.3]	2.41 [-1.32,5.34]	9.15 [2.64,14.14]	2.82 [-1.95,6.72]	10.46 [2.44,16.75]	1.33 [-2.81,4.46]	7.6 [-10.41,17.06]	0.11 [-6.54,5.15]
**9**	Brazil	6.88 [6.01,7.62]	2.46 [1.7,3.19]	8.3 [6.92,9.63]	2.72 [1.41,3.7]	6.07 [4.89,7]	2.49 [1.43,3.37]	29.39 [10.93,43.57]	0.63 [-0.28,1.4]	9.18 [-2.62,18.6]	0.55 [-6.77,7.87]
**South Africa**											
**10**	South Africa	8.62 [8,9.09]	1.55 [1.08,1.94]	7.01 [5.88,7.91]	1.14 [0.34,1.72]	8.03 [7.34,8.53]	3.31 [2.9,3.64]	10.61 [9.64,11.29]	0.87 [0.37,1.21]	3.56 [-0.79,5.67]	0.82 [-0.73,1.82]
**North Europe**											
**11**	Finland	3.85 [0.43,6.6]	3.05 [0.82,4.94]	5.68 [1.68,9.1]	3.74 [1.44,5.83]	3.94 [-0.45,7.59]	1.14 [-1.4,3.35]	6.18 [-2.95,12.88]	2.52 [-4.21,7.16]	-0.49 [-11.38,6.78]	3.38 [-2.27,7.93]
**12**	Estonia	7.89 [6.42,9.21]	2.42 [1.35,3.41]	8.43 [6.35,10.16]	2.1 [0.94,3.13]	7.05 [4.79,8.84]	2.64 [1.37,3.84]	6.46 [1.26,9.52]	6.07 [2.81,8.2]	-1.84 [-16.5,6.01]	4.5 [-2.23,10.09]
**13**	UK	9.02 [8.45,9.47]	1.63 [1.27,1.95]	10.33 [9.65,10.91]	1.5 [1.1,1.85]	6.71 [5.91,7.38]	1.73 [1.31,2.11]	5.15 [3.1,6.69]	3.05 [2.04,3.74]	1.4 [-1.71,3]	0.87 [-0.32,1.69]
**Central Europe**											
**14**	Switzerland	6.96 [5.41,8.29]	3.58 [2.36,4.57]	7.71 [5.49,9.5]	3.3 [1.97,4.31]	4.33 [1.98,6.29]	1.4 [-0.51,3.05]	5.61 [2.11,8.12]	3.88 [1.4,5.77]	4.32 [-1.93,7.69]	-0.73 [-5.21,1.98]
**15**	Moldova	12.32 [9.13,14.83]	5.41 [2.74,7.63]	12.16 [8.18,15.52]	3.24 [0.44,5.87]	10.99 [6.94,14.17]	6.35 [2.42,9.77]	NA	NA	NA	NA
**South Europe**											

**16**	Portugal	13.19 [12.03,14.16]	6.66 [6,7.24]	14.52 [12.59,16.08]	4.16 [3.14,5.05]	13.37 [12.11,14.5]	7.31 [6.67,7.89]	11.82 [9.4,13.88]	5.35 [4.04,6.39]	9.95 [4.31,13.56]	0.02 [-1.99,1.42]
**17**	Spain	11.32 [10.08,12.39]	3.99 [3.22,4.69]	11.73 [9.75,13.44]	3.54 [2.29,4.48]	11.03 [9.41,12.44]	3.63 [2.67,4.57]	8.35 [5.47,10.76]	3.55 [1.99,4.88]	6.82 [1.11,10.59]	0.63 [-1.53,2.18]
**18**	Italy	9.55 [7.16,11.62]	3.94 [2.37,5.3]	11.61 [7.78,14.44]	3.21 [1.36,4.84]	5.93 [1.39,9.15]	2.78 [0.03,5.02]	8.55 [2.96,12.85]	3.13 [0.06,5.64]	8.11 [-3.05,13.86]	0.16 [-2.4,2.21]
**19**	Cyprus	13.5 [10.55,15.8]	4.26 [2.71,5.6]	12.42 [8.22,15.64]	2.6 [0.88,4.01]	10.92 [6.9,14]	2.27 [-0.13,4.12]	11.2 [6.76,14.39]	1.53 [-0.78,3.36]	9.94 [-0.59,15.24]	0.19 [-1.6,1.71]
**Middle–East Asia**											
**20**	Iran	4.78 [2.73,6.74]	1.47 [-0.1,3]	3.74 [-0.04,6.69]	1.57 [-0.5,3.42]	4.82 [2.27,7.09]	0.69 [-0.16,1.45]	4.14 [-0.16,7.81]	2.32 [-0.11,4.15]	2.13 [-4.99,6.49]	3.24 [0.41,5.51]
**21**	Kuwait	12.11 [7.5,16.06]	0.01 [-1.95,1.68]	10 [3.73,14.97]	1.81 [-3.26,5.72]	14.49 [8.34,19.06]	0.46 [-3.69,4.05]	17.3 [8.8,23.31]	-0.38 [-5.37,2.73]	18.09 [-11.39,29.91]	-1.35 [-8.95,3.88]
**East Asia**											
**22**	South Korea	7.96 [7.18,8.61]	1.5 [0.93,2.03]	7.56 [5.95,8.77]	1.77 [0.88,2.64]	7.54 [6.56,8.33]	0.66 [0.25,1.07]	NA	NA	NA	NA
**23**	Japan	12.69 [12.4,12.95]	2.32 [2.05,2.54]	14.35 [13.89,14.72]	3.55 [3.17,3.85]	10.97 [10.62,11.27]	1.39 [1.17,1.59]	14.64 [14.15,15.06]	3.27 [2.91,3.58]	9.59 [8.55,10.16]	1.07 [0.29,1.66]
**24**	Taiwan	12.52 [10.24,14.5]	3.89 [1.84,5.96]	10.53 [6.78,13.82]	4.45 [1.18,7.1]	11 [7.14,13.76]	2.84 [-0.26,5.55]	12.44 [7.79,15.64]	4.4 [-0.06,7.67]	12.78 [-0.44,20.07]	-3.07 [-8.8,1.26]
**South East Asia**											
**25**	Thailand	6.43 [5.87,6.85]	0.71 [-0.03,1.27]	6.28 [5.36,6.97]	0.88 [-0.22,1.66]	8.06 [7.41,8.52]	0.09 [-0.79,0.82]	6.7 [5.57,7.3]	0.09 [-0.46,0.46]	5.6 [2.85,7.01]	-3.84 [-7.92,-1.62]
**26**	Philippines	3.32 [1.01,5.33]	3.89 [1.86,5.74]	2.66 [-0.43,4.87]	2.78 [0.08,5.02]	8.39 [6.26,10.16]	5.59 [3.15,7.65]	8.1 [5.43,10.48]	2.61 [-2.94,6.61]	4.16 [-7.09,9.32]	-5.27 [-18.5,2.67]
**27**	Vietnam	1.08 [-1.72,3.35]	1.7 [-5.09,6.77]	2.73 [-1.35,5.72]	0.32 [-8.86,6.59]	2.32 [-0.28,4.58]	0.46 [-8.98,7.42]	5.84 [1.52,9.09]	8.4 [-4.13,16.09]	NA	NA
**Total**											
	**Total**	9.09 [8.89,9.19]	2.22 [2.07,2.31]	8.6 [8.29,8.76]	2.33 [2.14,2.44]	9.02 [8.77,9.18]	1.64 [1.5,1.75]	12.8 [12.23,13.1]	2.63 [2.37,2.81]	6.87 [5.89,7.06]	0.84 [0.32,1.12]

Table S6. Excess deaths (per 1000 deaths) attributable all cold temperatures (below the minimum mortality temperature) and all hot temperatures (above the minimum mortality temperature)

		All CVD		Ischemic Heart Disease		Stroke		Heart Failure		Arrythmia	
	**Country**	**Cold**	**Heat**	**Cold**	**Heat**	**Cold**	**Heat**	**Cold**	**Heat**	**Cold**	**Heat**
**North America**											
**1**	Canada	107.37 [94.25,119.87]	2.1 [1.53,2.6]	100.26 [82.61,115]	2.01 [1.38,2.56]	119.73 [105.67,132.28]	2.21 [1.33,2.95]	132.32 [97.86,157.29]	3.13 [1.62,4.53]	66 [30.21,92.95]	3.65 [0.96,5.93]
**2**	US	80.59 [76.13,84.63]	2.69 [2.36,2.93]	84.09 [77.73,89.16]	2.75 [2.21,3.15]	76.32 [71.81,79.85]	2.31 [1.51,2.96]	109.95 [97.88,117.83]	0.25 [-0.35,0.71]	72.37 [60.72,79.97]	3.92 [2.81,4.59]
**Caribbean and Central America**											
**3**	Guatemala	63.76 [-4.6,125.05]	0.33 [-1.26,1.67]	29.18 [-61.11,107.87]	0.32 [-0.97,1.41]	48.71 [-31.3,115.48]	1.01 [-4.42,5.51]	20.08 [-7.57,45.31]	13.19 [-41.03,67.33]	35.84 [-90.58,141.98]	4.82 [-4.03,12.9]
**4**	Costa Rica	53.61 [-33.88,128.03]	0.28 [-2.3,2.25]	104.39 [7.39,179.76]	3.33 [-2.17,8.59]	11.72 [-12.99,34.07]	8.72 [-21.76,37.2]	41.99 [-24.67,105.82]	3.72 [-26.68,29.29]	NA	NA
**5**	Panama	38.42 [-26.37,98]	7.66 [-7.86,22.82]	50.23 [-43.76,135.54]	6.73 [-4.6,16.73]	44.76 [-18.32,100.8]	10.3 [-11.52,26.95]	NA	NA	NA	NA
**South America**											
**6**	Uruguay	88.32 [31.99,140.83]	9.24 [-0.05,17.89]	397.16 [44.01,695.91]	13.3 [-9.2,33.45]	314 [54.45,558.55]	37.41 [-15.8,79.64]	440.28 [-31.85,821.28]	20.29 [-6.03,40.47]	247.65 [-100.44,549.89]	14.01 [-19.41,41.17]
**7**	Ecuador	47.32 [14.32,76.46]	12.89 [2.28,23.25]	17.16 [-14.99,48.6]	19.25 [-11,45.55]	68.47 [18.75,112.66]	11.17 [2.55,19.16]	37.77 [-4.57,73.7]	9.4 [-35.46,43.39]	71.56 [-24.71,147.78]	5.01 [-6.03,13.18]
**8**	Paraguay	90.48 [37.42,132.46]	7.66 [-1.91,16.2]	91.01 [15.89,157.71]	6.72 [-3.62,15.35]	61.75 [1.94,118.06]	5.48 [-6.41,16.11]	96.27 [-28.47,209.09]	1.44 [-3.5,5.3]	82.83 [-14.45,163.45]	6.46 [-9.38,20.67]
**9**	Brazil	57.33 [45.35,69.4]	5.49 [3.42,7.33]	69.11 [50.4,86.76]	6.68 [3.1,9.78]	35.29 [23.36,46.77]	7.65 [3.1,11.64]	50.34 [25.88,70.7]	10.78 [-1.52,21.54]	67.88 [-19.07,145.3]	1.49 [-7.24,9.03]
**South Africa**											
**10**	South Africa	110.14 [102.55,116.46]	3.48 [2.63,4.24]	87.72 [71.93,99.94]	1.97 [0.59,3.08]	88.04 [79.42,95.7]	6.89 [5.85,7.81]	161.02 [138.55,178.21]	1.14 [0.47,1.56]	53.79 [20.76,78.15]	7.63 [3.69,10.83]
**North Europe**											
**11**	Finland	53.44 [-0.88,106.86]	3.7 [0.69,6.21]	72.2 [-2.41,139.23]	4.45 [1.68,7.04]	97.35 [26.67,163.7]	1.21 [-1.53,3.71]	116.17 [-17.61,225.69]	3.02 [-6,10.28]	62.71 [-53.82,158.87]	7.26 [-5.52,18.19]
**12**	Estonia	120.25 [98.44,140.87]	2.92 [1.49,4.25]	137.2 [104.64,165.91]	2.36 [0.87,3.55]	117.86 [85,144.67]	3.32 [1.37,4.91]	116.18 [55.75,167.75]	6.68 [2.91,9.61]	21.62 [-65.72,87.63]	8.57 [-4.69,20.47]
**13**	UK	109.12 [99.58,118.22]	1.8 [1.36,2.18]	126.13 [113.29,138.07]	1.6 [1.13,2.02]	86.33 [75.39,95.17]	2.5 [1.72,3.22]	71.89 [39.84,96.43]	3.48 [2.23,4.48]	51.46 [21.78,74.22]	4.8 [1.99,7]
**Central Europe**											
**14**	Switzerland	99.84 [72.48,124.28]	4.56 [2.89,6.1]	109.31 [77.13,142.06]	3.86 [2.39,5.15]	52.63 [23.97,79.08]	2.59 [-1.74,6.09]	94.6 [42.45,133.1]	8.56 [2.71,12.84]	112.24 [54.81,156.06]	1.34 [-4.97,6.31]
**15**	Moldova	160.02 [108.13,205.2]	7.83 [2.16,13.2]	151.93 [86.91,205.89]	4.93 [-1.06,10.18]	151.37 [88.92,207.28]	8.38 [0.95,14.6]	NA	NA	NA	NA
**South Europe**											
**16**	Portugal	126.18 [112.63,140.06]	12.02 [10.44,13.56]	164.24 [137.89,185.69]	6.35 [4.12,8.33]	112.35 [98.12,126.77]	15.31 [13.49,17.03]	140.51 [102.8,174.38]	7.25 [5.07,9.16]	111.78 [57.08,156.91]	1.66 [-1.5,4.43]

**17**	Spain	124.87 [108.9,139.27]	7.03 [4.96,9.14]	138.48 [109.11,161.32]	5.49 [3.15,7.84]	120.95 [101.08,141.26]	7.26 [4.6,9.57]	93.14 [44.56,132.71]	4.52 [2.26,6.35]	107.76 [61.17,148.61]	4.42 [0.22,8.33]
**18**	Italy	145.32 [113.07,176.23]	9.92 [5.4,14.08]	143.38 [89.18,185.62]	5.04 [0.46,9.05]	109.71 [66.38,147.95]	4.24 [-1.83,9.69]	142.05 [61.18,198.43]	1.77 [-1.15,4.19]	133.57 [70.72,186.27]	3.37 [-1.95,7.84]
**19**	Cyprus	106.61 [75.93,135.85]	9.89 [5.05,14.5]	141.33 [94.32,183.73]	7.99 [2.47,13.84]	60.13 [17.8,100.58]	4.98 [-2.4,12.2]	117.3 [21.63,198.18]	4.25 [-0.22,8.39]	104.09 [12.46,180.82]	2.72 [-3.73,9.5]
**Middle–East Asia**											
**20**	Iran	76.93 [41.68,111]	2.44 [-1.85,6.24]	51.59 [-1.16,101.49]	3.84 [-2.84,9.77]	132.57 [62.17,192.82]	0.74 [-0.24,1.57]	111.38 [-4.89,202.7]	2.58 [-0.29,4.81]	70.48 [-5.06,137.6]	8.43 [-1.45,17.11]
**21**	Kuwait	135.05 [15.4,238.17]	0.01 [-2.19,1.79]	112.13 [25.54,188.42]	5.98 [-5.55,16.51]	153.05 [58.92,233.96]	0.49 [-4.11,4.46]	203.32 [1.34,366.45]	-0.1 [-4.75,3]	184.83 [43.84,291.22]	0.44 [-15.91,14.43]
**East Asia**											
**22**	South Korea	143.28 [126.02,157.97]	1.72 [1.03,2.33]	101.42 [76.94,124.64]	2.21 [0.83,3.32]	143.18 [127.12,159.04]	0.72 [0.25,1.15]	NA	NA	NA	NA
**23**	Japan	191.36 [187.36,195.25]	4.05 [3.54,4.49]	224.22 [216.93,230.66]	5.98 [5.24,6.65]	157.93 [153.26,162.06]	2.39 [1.93,2.83]	220.8 [212.21,228.21]	6.28 [5.42,7.03]	168.24 [153.04,180.52]	2.15 [0.95,3.06]
**24**	Taiwan	109.3 [79.24,137.3]	7.67 [2.69,13.14]	94.55 [46.97,132.2]	8.06 [-0.04,14.95]	97.52 [58.55,133.25]	6.82 [-1.15,14.12]	121.31 [44.59,186.56]	5.62 [-0.73,10.85]	111.51 [36.61,168.31]	3.21 [-7.12,11.86]
**South East Asia**											
**25**	Thailand	78.13 [68.61,86.48]	0.9 [-0.17,1.75]	52.28 [40.45,62.66]	2.89 [0.68,4.68]	82.15 [72.82,90.77]	1.66 [-0.18,3.11]	60.18 [31.48,81.67]	0.65 [-0.53,1.52]	59.32 [27.99,83.69]	5.77 [-1.23,10.38]
**26**	Philippines	20.37 [9.63,30.29]	10.45 [3.51,17.74]	12.05 [0.84,23.29]	15.52 [1.07,28.76]	41.92 [24.53,58.41]	13.63 [6.81,19.26]	27.41 [10.82,41.53]	19.02 [-12.09,44.58]	56.77 [-14.59,118.52]	5.36 [-9.1,17.2]
**27**	Vietnam	6.33 [-12.2,23.92]	4.46 [-21.54,26.59]	15.79 [-30.95,62.28]	6.44 [-12.46,25.24]	12.91 [-4.93,28.36]	9.98 [-22.32,38.08]	58.88 [-74.85,169.84]	9.53 [-5.18,19.06]	NA	NA
**Total**											
	**Total**	127.08 [124.52,129.1]	3.5 [3.25,3.7]	118.9 [114.83,122.07]	3.55 [3.17,3.81]	124.37 [121.36,126.63]	3.15 [2.78,3.45]	186.36 [179.14,191.25]	5.05 [4.38,5.58]	122.02 [111.57,128.43]	3.12 [2.23,3.59]

Table S7. Top five cities with highest relative risk of cause-specific CVD death, stratified by quartiles of GDP per capita

**Extreme Cold (1st percentile vs. MMT)**				**Extreme Heat (99th percentile vs. MMT)**		
Country	City	RR		Country	City	RR
**All-Cause Cardiovascular**						
**GDP per capita - 1st Quartile (lowest)**						
Moldova	Chisinau	1.706 [1.444,2.016]		Moldova	Chisinau	1.253 [1.103,1.423]
Paraguay	Asuncion	1.535 [1.24,1.9]		Philippines	Cebu	1.184 [0.997,1.406]
Thailand	Nakhon Phanom	1.435 [1.077,1.911]		Paraguay	Asuncion	1.158 [0.996,1.348]
Thailand	Prachuap Khiri Khan	1.399 [1.096,1.785]		Thailand	Yala	1.152 [0.727,1.825]
Thailand	Lop Buri	1.394 [1.142,1.701]		Philippines	Manila	1.14 [1,1.3]
**GDP per capita - 2nd Quartile**						
Taiwan	Taipei	1.728 [1.472,2.028]		Taiwan	Taipei	1.312 [1.064,1.618]
Brazil	Florianopolis	1.583 [1.327,1.889]		Ecuador	Guayaquil	1.253 [1.081,1.452]
Taiwan	Taichung	1.582 [1.275,1.962]		Uruguay	Montevideo	1.253 [1.062,1.479]
South Africa	Amajuba	1.543 [1.272,1.871]		South Africa	Joe Gqabi	1.246 [1.004,1.545]
Taiwan	Kaohsiung	1.519 [1.275,1.809]		Brazil	Florianopolis	1.243 [1.083,1.427]
**GDP per capita - 3rd Quartile**						
Cyprus	Nicosia	1.702 [1.304,2.221]		Portugal	Castelo Branco	1.556 [1.358,1.782]
Cyprus	Limassol	1.657 [1.342,2.047]		Portugal	Lisboa	1.485 [1.382,1.596]
Cyprus	Larnaca	1.647 [1.222,2.218]		Portugal	Coimbra	1.477 [1.332,1.637]
Portugal	Lisboa	1.602 [1.49,1.723]		Portugal	Beja	1.463 [1.326,1.615]
Portugal	Castelo Branco	1.601 [1.405,1.825]		Italy	Civitavecchia	1.433 [1.099,1.868]
**GDP per capita - 4th Quartile (highest)**						
Japan	Nagasaki	1.849 [1.702,2.009]		Switzerland	Ticino	1.316 [1.077,1.607]
Japan	Shizuoka	1.836 [1.69,1.995]		Switzerland	St. Gallen	1.263 [1.048,1.521]
Japan	Ehime	1.808 [1.655,1.976]		US	New York, NY	1.245 [1.171,1.323]
Japan	Kagoshima	1.798 [1.657,1.95]		Japan	Kagawa	1.232 [1.102,1.376]
Japan	Tochigi	1.773 [1.616,1.946]		US	Chicago, IL	1.23 [1.162,1.303]
**Ischemic Heart Disease**						
**GDP per capita - 1st Quartile (lowest)**						
Moldova	Chisinau	1.67 [1.357,2.056]		Moldova	Chisinau	1.143 [1.006,1.299]
Thailand	Nakhon Phanom	1.565 [1.076,2.276]		Philippines	Cebu	1.142 [0.941,1.385]
Paraguay	Asuncion	1.529 [1.136,2.057]		Philippines	Manila	1.115 [0.966,1.288]
Thailand	Prachuap Khiri Khan	1.486 [1.08,2.044]		Paraguay	Asuncion	1.11 [0.963,1.281]
Thailand	Lop Buri	1.45 [1.111,1.893]		Philippines	Davao	1.095 [0.93,1.291]
**GDP per capita - 2nd Quartile**						

Brazil	Florianopolis	1.573 [1.255,1.972]		Taiwan	Taipei	1.348 [1.02,1.781]
Brazil	Porto Alegre	1.563 [1.33,1.836]		Brazil	Florianopolis	1.19 [1.009,1.403]
Costa Rica	San José (CR)	1.556 [1.186,2.041]		Brazil	Sao Paulo	1.184 [1.078,1.3]
Taiwan	Taipei	1.556 [1.214,1.994]		Brazil	Porto Alegre	1.168 [1.053,1.297]
Taiwan	Taichung	1.541 [1.15,2.065]		Ecuador	Guayaquil	1.136 [0.984,1.312]
**GDP per capita - 3rd Quartile**						
Portugal	Castelo Branco	1.739 [1.439,2.103]		Italy	Civitavecchia	1.305 [0.987,1.726]
Portugal	Porto	1.703 [1.465,1.98]		Portugal	Lisboa	1.285 [1.172,1.409]
Portugal	Lisboa	1.686 [1.507,1.886]		Portugal	Castelo Branco	1.263 [1.071,1.489]
Portugal	Coimbra	1.671 [1.386,2.015]		Spain	Malaga	1.248 [1.063,1.466]
Portugal	Faro	1.657 [1.402,1.958]		Portugal	Porto	1.241 [1.092,1.409]
**GDP per capita - 4th Quartile (highest)**						
Japan	Shizuoka	2.071 [1.813,2.366]		Japan	Osaka	1.339 [1.237,1.449]
Japan	Saitama	2.01 [1.792,2.254]		Japan	Kagawa	1.277 [1.097,1.488]
Japan	Tochigi	2.006 [1.736,2.318]		Switzerland	St. Gallen	1.271 [1.038,1.557]
Japan	Kochi	1.996 [1.69,2.357]		Japan	Ibaraki	1.231 [1.073,1.413]
Japan	Kagoshima	1.983 [1.738,2.262]		US	New York, NY	1.221 [1.146,1.302]
**Stroke**						
**GDP per capita - 1st Quartile (lowest)**						
Thailand	Nakhon Phanom	1.72 [1.268,2.334]		Philippines	Cebu	1.27 [1.035,1.557]
Thailand	Prachuap Khiri Khan	1.661 [1.278,2.159]		Moldova	Chisinau	1.266 [1.067,1.503]
Moldova	Chisinau	1.611 [1.303,1.992]		Philippines	Manila	1.235 [1.046,1.459]
Thailand	Pattani	1.556 [1.253,1.933]		Philippines	Davao	1.198 [1.019,1.408]
Thailand	Ubon Ratchathani	1.546 [1.281,1.866]		Philippines	Quezon	1.165 [1.031,1.316]
**GDP per capita - 2nd Quartile**						
Ecuador	Guayaquil	1.631 [1.205,2.207]		South Africa	Joe Gqabi	1.492 [1.234,1.805]
Taiwan	Taipei	1.584 [1.272,1.973]		South Africa	Mopani	1.345 [1.157,1.563]
Brazil	Florianopolis	1.532 [1.295,1.813]		South Africa	Vhembe	1.341 [1.147,1.569]
South Africa	Vhembe	1.532 [1.253,1.873]		South Africa	Overberg	1.336 [1.155,1.544]
South Africa	John Taolo Gaetsewe	1.488 [1.199,1.848]		South Africa	Siyanda	1.33 [1.143,1.547]
**GDP per capita - 3rd Quartile**						
Kuwait	Kuwait	1.665 [1.313,2.112]		Portugal	Castelo Branco	1.661 [1.495,1.845]
Portugal	Castelo Branco	1.648 [1.441,1.884]		Portugal	Porto	1.569 [1.428,1.725]
Portugal	Coimbra	1.608 [1.416,1.825]		Portugal	Coimbra	1.558 [1.423,1.707]
Spain	Madrid	1.608 [1.385,1.865]		Portugal	Beja	1.501 [1.379,1.632]

Portugal	Porto	1.591 [1.432,1.768]		Portugal	Lisboa	1.464 [1.354,1.583]
**GDP per capita - 4th Quartile (highest)**						
Japan	Chiba	1.618 [1.47,1.78]		Canada	Calgary	1.161 [1.03,1.308]
Japan	Nagasaki	1.618 [1.473,1.778]		Switzerland	Ticino	1.151 [0.863,1.536]
Japan	Shizuoka	1.605 [1.458,1.768]		US	Albany, NY	1.143 [1.024,1.277]
Japan	Kyoto	1.602 [1.452,1.767]		Japan	Niigata	1.131 [1.052,1.216]
Japan	Okinawa	1.597 [1.392,1.832]		UK	Northampton	1.127 [1.023,1.241]
**Heart Failure**						
**GDP per capita - 1st Quartile (lowest)**						
Vietnam	Ho Chi Minh City	1.493 [1.12,1.99]		Vietnam	Ho Chi Minh City	1.734 [0.812,3.704]
Paraguay	Asuncion	1.467 [1.063,2.023]		Iran	Mashhad	1.262 [1.006,1.584]
Thailand	Prachuap Khiri Khan	1.453 [1.045,2.02]		Philippines	Cebu	1.096 [0.783,1.533]
Philippines	Manila	1.429 [1.136,1.796]		Philippines	Davao	1.087 [0.838,1.408]
Thailand	Uttaradit	1.425 [1.077,1.885]		Philippines	Manila	1.068 [0.827,1.38]
**GDP per capita - 2nd Quartile**						
South Africa	Siyanda	1.743 [1.353,2.244]		Taiwan	Taipei	1.504 [0.886,2.553]
Taiwan	Taipei	1.727 [1.3,2.293]		South Africa	Joe Gqabi	1.341 [0.974,1.847]
South Africa	Vhembe	1.695 [1.301,2.209]		Uruguay	Montevideo	1.216 [0.954,1.551]
South Africa	Dr Ruth Segomotsi Mompati	1.692 [1.319,2.17]		Taiwan	Taichung	1.182 [0.943,1.48]
South Africa	Ngaka Modiri Molema	1.661 [1.313,2.101]		South Africa	Overberg	1.173 [0.956,1.439]
**GDP per capita - 3rd Quartile**						
Kuwait	Kuwait	1.868 [1.282,2.72]		Portugal	Castelo Branco	1.527 [1.243,1.875]
Cyprus	Limassol	1.571 [1.152,2.143]		Portugal	Porto	1.419 [1.183,1.702]
Cyprus	Nicosia	1.557 [1.114,2.177]		Italy	Civitavecchia	1.408 [0.883,2.244]
Portugal	Castelo Branco	1.531 [1.223,1.917]		Portugal	Coimbra	1.399 [1.176,1.665]
Cyprus	Ammochostos	1.514 [1.08,2.123]		Portugal	Beja	1.338 [1.15,1.556]
**GDP per capita - 4th Quartile (highest)**						
Japan	Kagoshima	2.112 [1.826,2.444]		Japan	Iwate	1.379 [1.159,1.64]
Japan	Yamanashi	2.022 [1.674,2.442]		Japan	Fukushima	1.313 [1.136,1.518]
Japan	Shizuoka	2.012 [1.761,2.298]		Japan	Aomori	1.283 [1.128,1.459]
Japan	Ibaraki	1.997 [1.736,2.299]		UK	Northampton	1.279 [1.028,1.592]
Japan	Ehime	1.986 [1.717,2.298]		Switzerland	St. Gallen	1.277 [0.885,1.843]
**Arrhythmia**						
**GDP per capita - 1st Quartile (lowest)**						
Paraguay	Asuncion	1.354 [0.803,2.283]		Iran	Mashhad	1.191 [0.981,1.447]
Thailand	Bangkok	1.305 [0.976,1.746]		Iran	Tehran	1.116 [0.975,1.277]

Thailand	Udon Thani	1.304 [0.868,1.957]		Philippines	Quezon	1.088 [0.902,1.312]
Thailand	Nakhon Sawan	1.293 [0.949,1.761]		Thailand	Chanthaburi	1.08 [0.908,1.283]
Thailand	Chaiyaphum	1.292 [0.871,1.915]		Guatemala	Guatemala	1.079 [0.95,1.226]
**GDP per capita - 2nd Quartile**						
Taiwan	Taipei	1.594 [0.978,2.597]		South Africa	Gert Sibande	1.095 [0.93,1.289]
Ecuador	Guayaquil	1.522 [0.713,3.247]		South Africa	Ekurhuleni	1.084 [0.936,1.256]
Taiwan	Taichung	1.392 [0.775,2.499]		South Africa	uThukela	1.08 [0.911,1.281]
Taiwan	Kaohsiung	1.357 [0.989,1.862]		Ecuador	Quito	1.079 [0.911,1.279]
South Africa	West Coast	1.348 [0.763,2.381]		South Africa	uMgungundlovu	1.076 [0.894,1.296]
**GDP per capita - 3rd Quartile**						
Kuwait	Kuwait	1.769 [0.95,3.293]		Estonia	Tartumaa	1.143 [0.928,1.408]
Cyprus	Nicosia	1.495 [0.78,2.865]		Estonia	Harjumaa	1.122 [0.948,1.329]
Cyprus	Limassol	1.47 [0.956,2.26]		Spain	Sevilla	1.056 [0.948,1.177]
Portugal	Beja	1.422 [1.018,1.987]		Spain	Madrid	1.055 [0.921,1.209]
Portugal	Lisboa	1.414 [1.06,1.887]		Portugal	Coimbra	1.046 [0.882,1.242]
**GDP per capita - 4th Quartile (highest)**						
Japan	Fukushima	2.142 [1.453,3.158]		Japan	Miyagi	1.184 [1.012,1.386]
Japan	Aichi	2.085 [1.557,2.792]		Japan	Iwate	1.183 [0.951,1.47]
Japan	Okinawa	2.045 [1.379,3.032]		Canada	Thunder Bay	1.153 [0.934,1.423]
Japan	Yamagata	1.972 [1.254,3.101]		Canada	Edmonton	1.13 [0.958,1.334]
Japan	Tochigi	1.949 [1.323,2.872]		US	Flint, MI	1.13 [0.91,1.404]

Gross Domestic Product (GDP) per capita quartiles; **1st quartile (lowest):** Vietnam, Philippines, Moldova, Thailand, Guatemala, Paraguay and Iran; **2nd quartile:** Ecuador, South Africa, Brazil, Costa Rica, Panama, Uruguay and Taiwan; **3rd quartile:** Estonia, Portugal, Cyprus, Kuwait, Italy, Spain and South Korea; **4th quartile (highest):** Japan, UK, Canada, US, Finland and Switzerland.

Table S8. Heterogeneity parameters in meta regression models

Choice	All CVD	IHD	Stroke	Heart Failure	Arrhythmia
**Intercept Only**	Q-test *p*<0.001<br>I^2 = 55.0%	Q-test *p*<0.001<br>I^2 = 36.9%	Q-test *p*<0.001<br>I^2 = 23.7%	Q-test *p*<0.001<br>I^2 = 21.9%	Q-test *p*=0.037<br>I^2 = 5.2%
**Main Model:**<br>Random Effects:<br>Level 1: cities<br>Level 2: climate zones within countries<br>Fixed Effects:<br>Mean summer temperature + Mean winter temperature + Country GDP per capita	Q-test *p*<0.001<br>I^2 = 50.0%	Q-test *p*<0.001<br>I^2 = 33.5%	Q-test *p*<0.001<br>I^2 = 17.6%	Q-test *p*<0.001<br>I^2 = 17.9%	Q-test *p*=0.062<br>I^2 = 4.5%

Table S9. Sensitivity Analyses and effect estimates for all-cause cardiovascular mortality

Choice	# of locations	All-Cause Cardiovascular Death			
		Relative Risk		Excess Deaths (per 1000 deaths)	
		Cold (1st vs. MMT)	Heat (99th vs. MMT)	Cold Range (2.5th and below)	Hot Range (97.5th and above)
**Modelling choices**					
Main analysis	567	1.323	1.105	9.09	2.22
21 lag days for temperature	567	1.378	1.089	9.72	2.00
4 df for temperature at the 10th, 50th, 75th and 90th percentiles	567	1.330	1.110	9.13	2.25
5 df for temperature at the 5th, 25th, 50th, 75th and 95th percentiles	567	1.339	1.099	9.15	2.18
Adjusting for long-term trend (natural spline 1 df/decade)	567	1.329	1.104	9.19	2.25
Adjusting for heatwave indicator (any 2 consecutive days > 95th percentile)	567	1.331	1.095	9.16	2.25
Adjusting for heatwave indicator (any 2 consecutive days > 99th percentile)	567	1.328	1.105	9.17	2.22
Adjusting for inter-day temperature variability	567	1.327	1.108	9.15	2.27
**Further Adjustments**					
Main analysis *Restricted to **RH** locations*	430	1.286	1.078	9.12	2.21
Control for **RH** *(24hr mean – Linear – lag0)*	430	1.289	1.076	9.13	2.22
Main analysis *Restricted to **ozone** locations*	308	1.440	1.113	8.09	2.47
Control for **ozone** *(8hr max – Linear – lag01)*	308	1.445	1.107	8.26	2.23
Main analysis *Restricted to **NO_2** locations*	301	1.401	1.113	8.32	2.55

Control for **NO_2** *(24hr mean – Linear – lag01)*	301	1.395	1.114	8.33	2.58
Main analysis *Restricted to **PM_{10}** locations*	234	1.399	1.111	10.05	2.93
Control for **PM_{10}** *(24hr mean – Linear – lag01)*	234	1.385	1.112	9.73	2.85
Main analysis *Restricted to **$PM_{2.5}$** locations*	230	1.388	1.126	9.10	2.99
Control for **$PM_{2.5}$** *(24hr mean – Linear – lag01)*	230	1.395	1.077	8.85	2.47

The main analysis includes temperature modelling using quadratic b-spline with 3 internal knots placed at the 10th, 75th, and 90th percentiles and a lag period of 14 days with no adjustment to other environmental exposures. Additionally, all main analyses include three fixed meta predictors: mean summer temperature, mean winter temperature and GDP per capita, as well as random effects for cities within country-specific climate zones.

Figure S1. Distribution of ambient temperatures in all 27 countries. Minimum mortality temperatures (for all-cause cardiovascular mortality) are shown in vertical dotted lines.

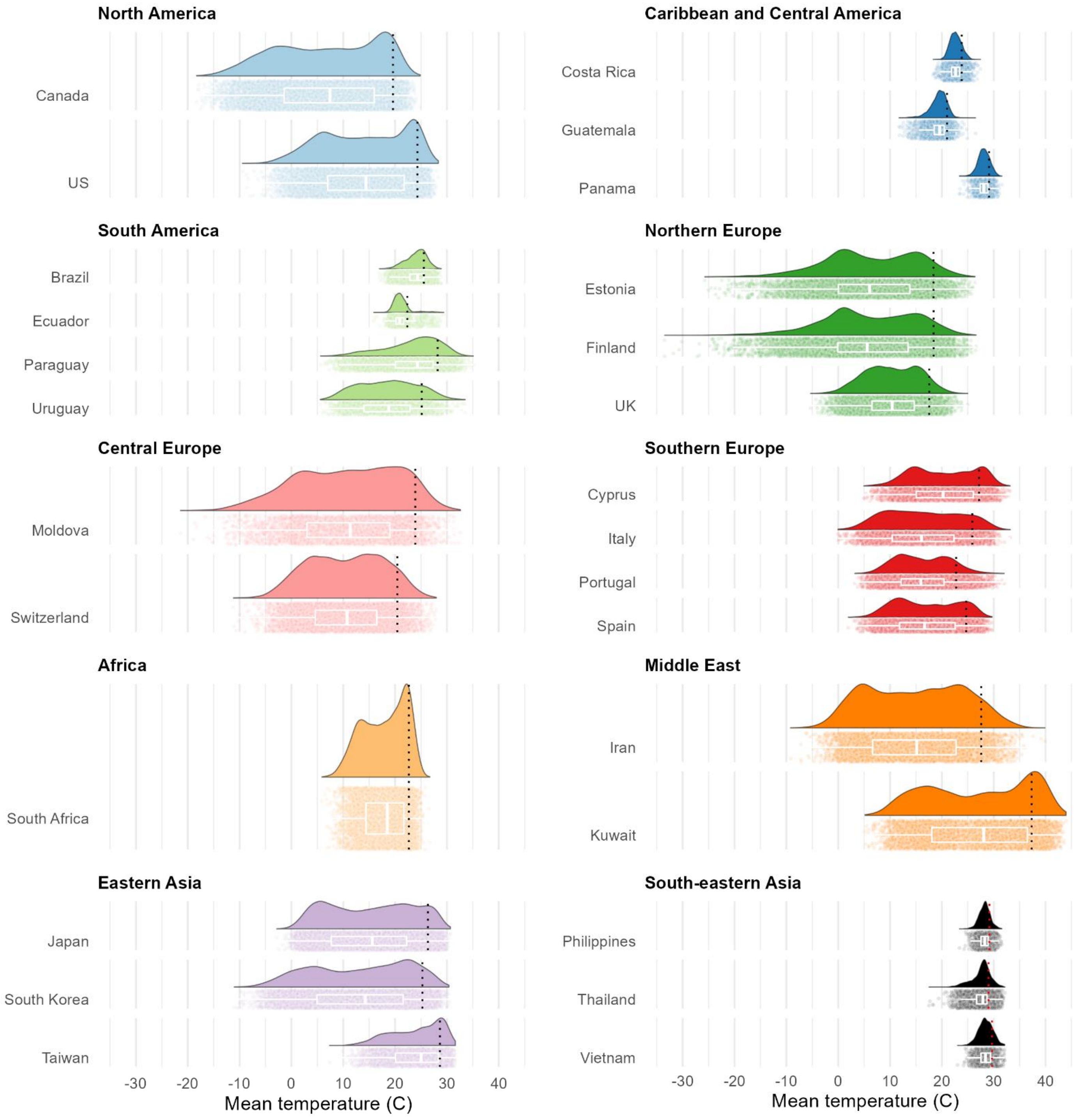

Figure S2. Pooled exposure-response relationships between temperature (in the absolute scale; °C) and relative risk (RR) of different causes of CVD mortality

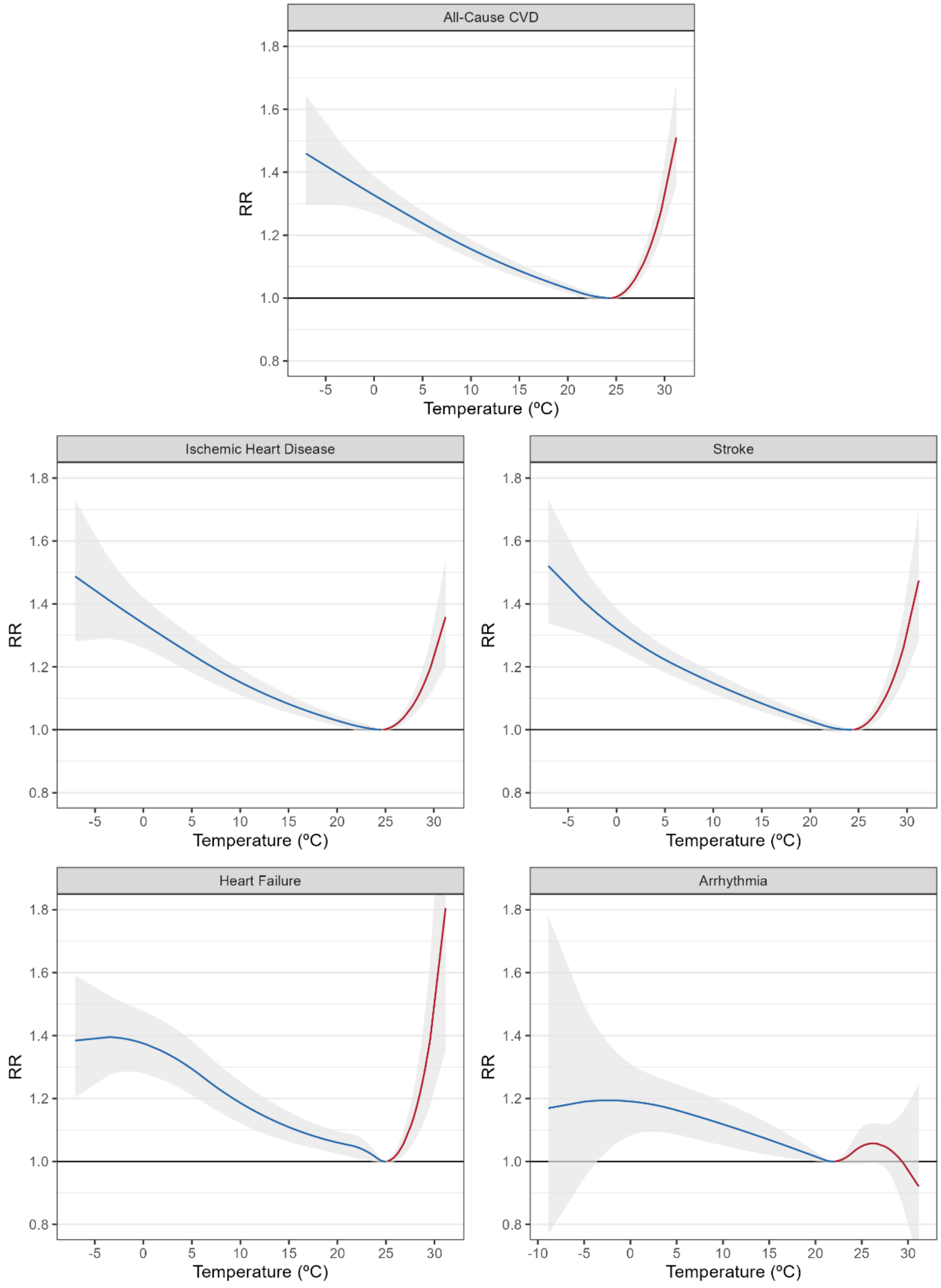

Figure S3. Exposure-response relationships between temperature (in the absolute scale; °C) and relative risk (RR) of all-cause CVD mortality in selected 12 cities around the world.

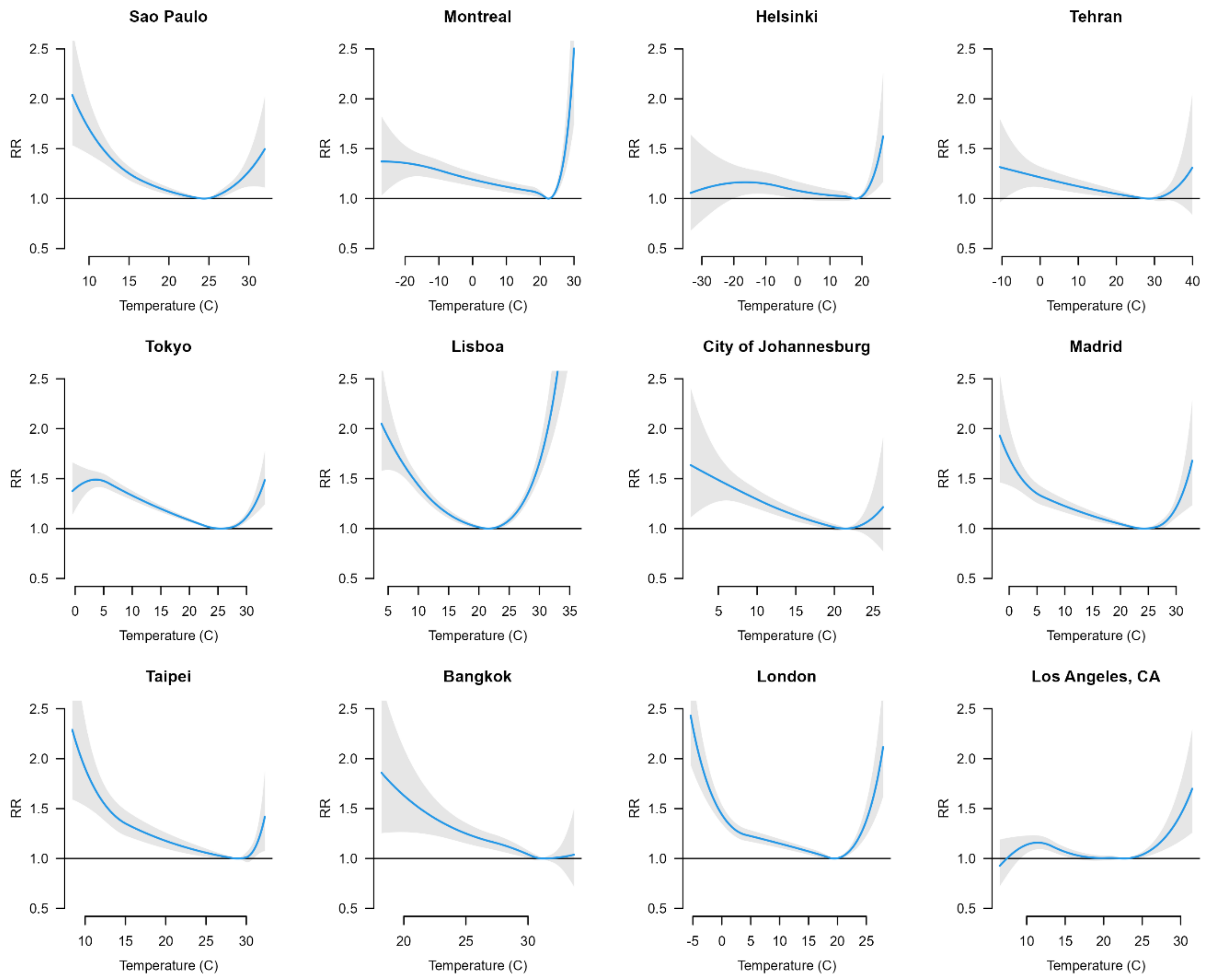

Figure S4. Pooled exposure-response relationships between temperature percentiles and relative risk (RR) of different causes of CVD mortality with stratification at the 25th percentile and 75th percentile of country-level gross domestic product (GDP) per capita.

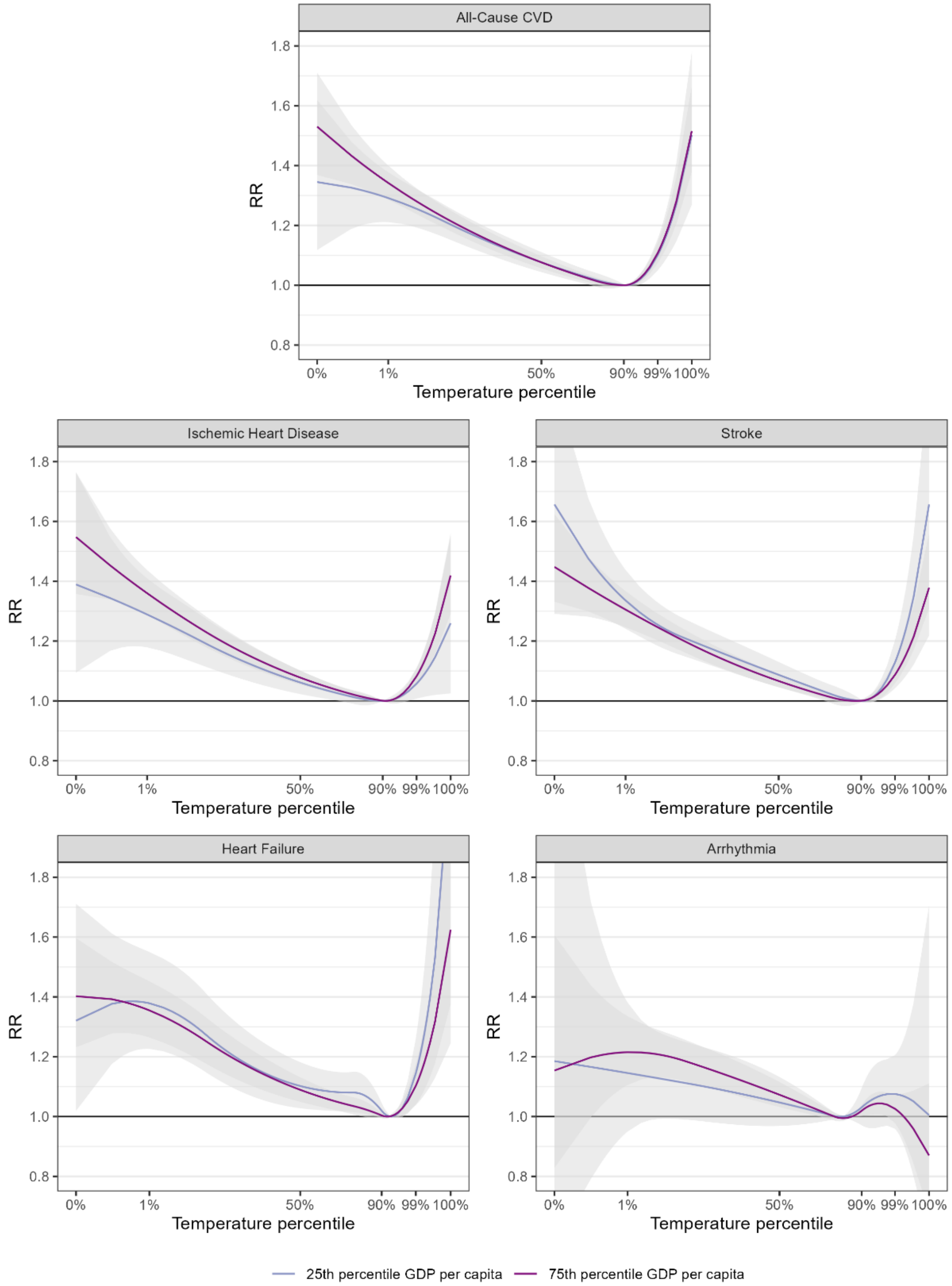

Figure S5. Pooled exposure-response relationships between temperature percentiles and relative risk (RR) of different causes of CVD mortality with stratification at the 25th percentile and 75th percentile of city-specific mean winter temperature.

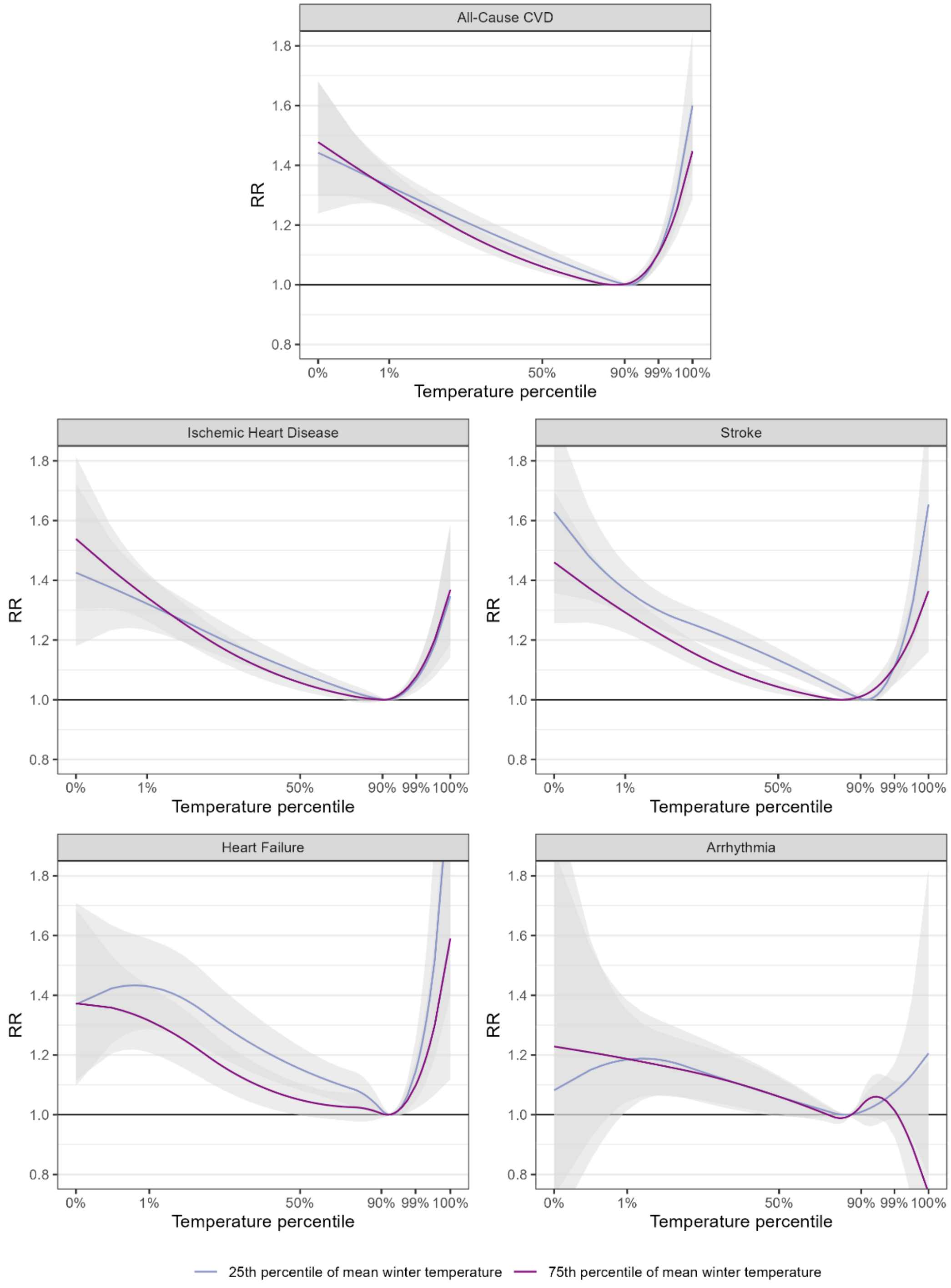

Figure S6. Pooled exposure-response relationships between temperature percentiles and relative risk (RR) of different causes of CVD mortality with stratification at the 25th percentile and 75th percentile of city-specific mean summer temperature.

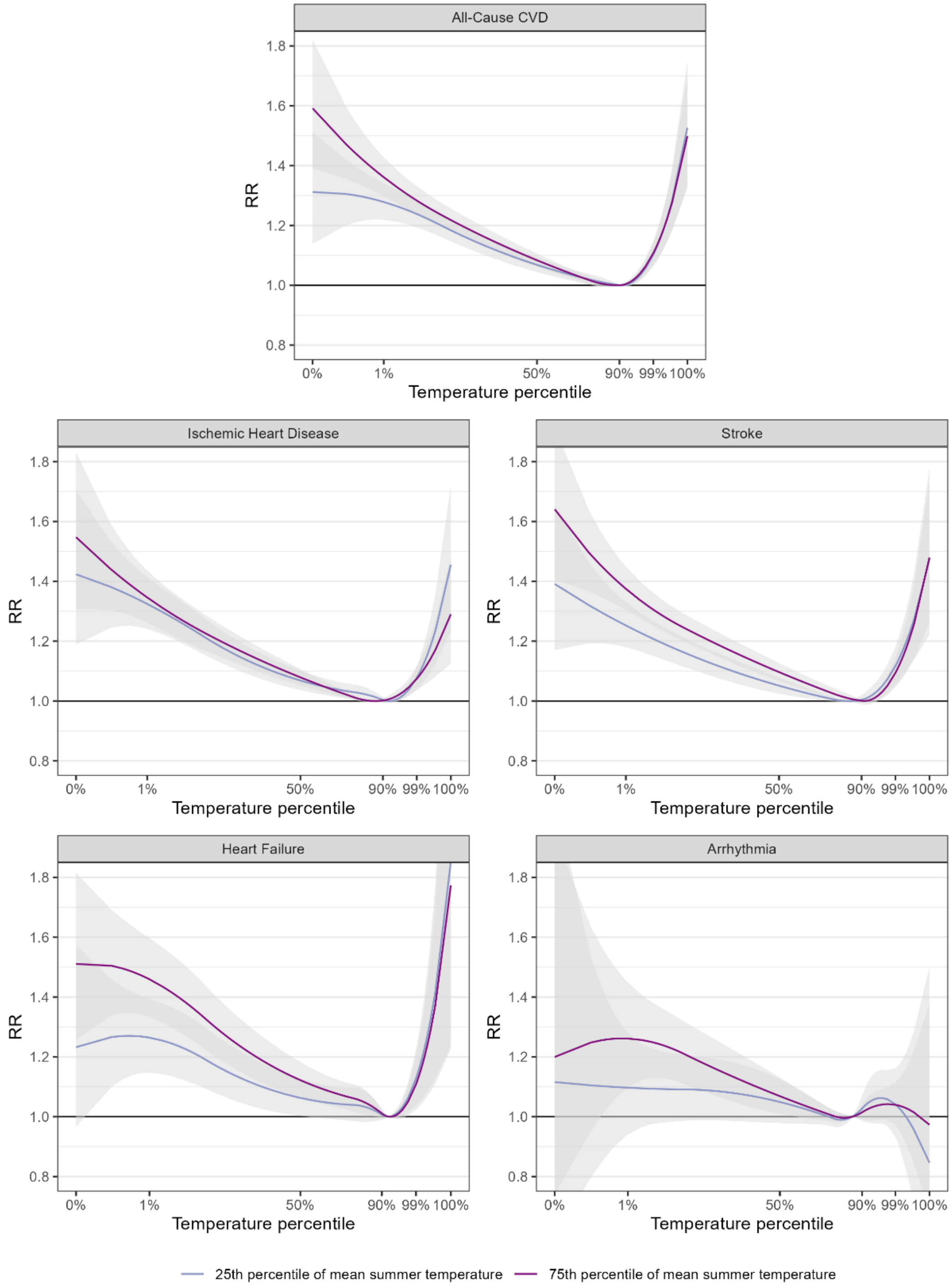

Supplement: Supplementary file 1 [file cir-147-35-s001.pdf]
